# New mechanistic insights of anti-obesity by sleeve gastrectomy-altered gut microbiota and lipid metabolism

**DOI:** 10.3389/fendo.2024.1338147

**Published:** 2024-02-02

**Authors:** Chuxuan Liu, Qian Xu, Shuohui Dong, Huanxin Ding, Bingjun Li, Dexu Zhang, Yongjuan Liang, Linchuan Li, Qiaoran Liu, Yugang Cheng, Jing Wu, Jiankang Zhu, Mingwei Zhong, Yihai Cao, Guangyong Zhang

**Affiliations:** ^1^Department of General Surgery, The First Affiliated Hospital of Shandong First Medical University, Jinan, Shandong, China; ^2^Department of General Surgery, Qilu Hospital, Shandong University, Jinan, Shandong, China; ^3^Department of Clinical Pharmacy, The First Affiliated Hospital of Shandong First Medical University, Jinan, Shandong, China; ^4^Department of Microbiology, Tumor and Cell Biology, Karolinska Institute, Stockholm, Sweden

**Keywords:** sleeve gastrectomy, obesity, microbiota, metagenome, metabolome

## Abstract

**Background:**

The obesity epidemic has been on the rise due to changes in living standards and lifestyles. To combat this issue, sleeve gastrectomy (SG) has emerged as a prominent bariatric surgery technique, offering substantial weight reduction. Nevertheless, the mechanisms that underlie SG-related bodyweight loss are not fully understood.

**Methods:**

In this study, we conducted a collection of preoperative and 3-month postoperative serum and fecal samples from patients who underwent laparoscopic SG at the First Affiliated Hospital of Shandong First Medical University (Jinan, China). Here, we took an unbiased approach of multi-omics to investigate the role of SG-altered gut microbiota in anti-obesity of these patients. Non-target metabolome sequencing was performed using the fecal and serum samples.

**Results:**

Our data show that SG markedly increased microbiota diversity and Rikenellaceae, *Alistipes*, *Parabacteroides*, Bactreoidales, and Enterobacteraies robustly increased. These compositional changes were positively correlated with lipid metabolites, including sphingolipids, glycerophospholipids, and unsaturated fatty acids. Increases of Rikenellaceae, *Alistipes*, and *Parabacteroide* were reversely correlated with body mass index (BMI).

**Conclusion:**

In conclusion, our findings provide evidence that SG induces significant alterations in the abundances of Rikenellaceae, *Alistipes*, *Parabacteroides*, and Bacteroidales, as well as changes in lipid metabolism-related metabolites. Importantly, these changes were found to be closely linked to the alleviation of obesity. On the basis of these findings, we have identified a number of microbiotas that could be potential targets for treatment of obesity.

## 1 Introduction

The prevalence of obesity has been steadily increasing over the past half century (1). Notably, the number of people with obesity in China grew threefold between 2004 and 2018; the mean body mass index (BMI) increased from 22.7 to 24.4 kg/m^2^ (2). Obesity contributes to numerous metabolic diseases, such as nonalcoholic fatty liver disease (NAFLD), type 2 diabetes mellitus (T2DM), cardiovascular disease, hypertension, coronary heart disease, depression, and cancer (3). A sedentary lifestyle, minimal exercise, and excessive energy intake are the main causes of obesity; obesity-related genes also constitute important genetic factors (4). Effective alleviation and reversal of obesity, as well as prevention of its complications and potential damaging effects, have received considerable public health interest.

The gut microbiota is the largest microbial community in the human body (5). At the genetic level, it contains approximately 30-fold more genes than the human genome (6), and it is closely associated with normal physiological functions of the human body (7). Recent studies have shown that changes in gut microbiota are strongly associated with the onset of obesity and obesity-related complications (e.g., reduced intestinal microbiota richness; reductions in Clostridiales, Deltaproteobacteria, and Pasteurellales; and an associated increase in Burkholderiales) in patients with obesity (8). The use of oral antibiotics may increase obesity risk (9), whereas fecal microbiota transplantation from lean donors can improve the metabolic status of obese patients and attenuate obesity-related metabolic diseases such as insulin resistance (10); these findings indicate that the gut microbiota plays an important role in obesity onset and progression. *Dysosmobacter welbionis*, a newly discovered gut probiotic, can effectively alleviate obesity, insulin resistance, and inflammation in adipose tissue, thereby improving host metabolic status (11). The specific mechanism of its effects may be closely associated with the small molecule metabolites it produces during fermentation, which can influence the local gut environment and systemic metabolic status (12). Short-chain fatty acids in the gut environment can regulate the progression of obesity through various effects on appetite and the systemic metabolic profile (13). The gut microbiota also has important effects on the production, transportation, and enterohepatic circulation of bile acids, which are important small molecule metabolites (14). Thus, targeted therapy against gut microbiota is gradually becoming an important intervention for obesity and obesity-related diseases.

Bariatric surgery, one of the most effective treatments for obesity and hyperglycemia, has substantial effects on metabolic status; it can alleviate and prevent obesity-related diseases such as T2DM and NAFLD (15, 16). Sleeve gastrectomy (SG), a common bariatric surgery approach, is widely used as treatment for obesity because of its simplicity, robust weight reduction effect, and low malnutrition risk (17, 18). The substantial weight-loss benefits of SG do not solely depend on gastric volume reduction; this surgical treatment can reorganize systemic endocrine functioning to improve glucose–lipid metabolism by regulating hormone levels, appetite, and other factors (19, 20). However, the details of the mechanism require further investigation. There is evidence that SG also influences gut microbiota composition and function, in a manner closely associated with its apparent therapeutic benefits (21). However, only a few studies have investigated the effects of SG on gut microbiota, mainly via 16S ribosomal RNA gene amplicon sequencing; additional research is needed concerning the precision and depth of its effects on microbial communities (22). Whole metagenome shotgun sequencing, a new technique that allows all microbial genomes in a sample to be sequenced, can localize bacteria to the species level and facilitate functional annotation; these results cannot be achieved using conventional 16S rDNA amplicon sequencing (23). Because of dietary and lifestyle differences among countries and regions, the gut microbiota displays substantial geographical variation. Although China has a large population, there have been few studies regarding the effects of SG on the gut microbiota and metabolites of Chinese obese patients. Here, we used whole metagenome shotgun sequencing, in combination with non-targeted metabolomic profiling, to elucidate the therapeutic mechanisms of SG from a gut microbiota perspective, identify biomarkers of SG, and provide new insights for obesity treatment.

## 2 Methods

### 2.1 Participants and sample collection

From September 2021 to May 2022, this study recruited obese patients who underwent laparoscopic SG at the First Affiliated Hospital of Shandong First Medical University (Jinan, China). None of the obese patients participating in this research had a drug history or medication withdrawal. Inclusion criteria were (1) a diagnosis of morbid obesity, (2) unsatisfactory results despite lifestyle and medical therapies, and (3) age 18–65 years. Exclusion criteria were (1) serious complications (e.g., poor cardiopulmonary function or any vital organ dysfunction), (2) cognitive impairment or intellectual disability, and/or (3) contraindications to laparoscopic SG or general anesthesia. Participants’ anthropometric data, serum samples, and fecal samples were collected (after an overnight fast) before surgery and 3 months after laparoscopic SG. After collection, serum and fecal samples were frozen at -80°C until analysis.

### 2.2 Operative procedure

After the induction of general anesthesia, each patient was placed in the supine position and a pneumoperitoneum was created at 12 mmHg with a Veress needle. A 10-mm trocar was placed for insertion of the 30° laparoscope, and four 5-mm trocars were inserted. The gastrocolic and gastrosplenic ligaments were dissected along the greater curvature of the stomach; dissection was continued proximally to the angle of His. Subsequently, excess gastric body and fundus were removed along the greater curvature of the stomach at 4 cm from the pylorus. Barbed sutures were used for continuous suturing to reinforce the cutting line. Next, a drainage tube was placed in the abdominal cavity. Finally, all trocars were removed, and the abdomen was deflated.

### 2.3 Whole metagenome shotgun sequencing

#### 2.3.1 Extraction of microbial DNA

Microbial DNA was extracted from fecal samples using the E.Z.N.A.^®^ Stool DNA Kit (D4015-02, Omega, Inc., USA), in accordance with the manufacturer’s instructions. This reagent, specifically formulated for the extraction of DNA from minimal sample sizes, has proven to be highly efficient in extracting DNA from a wide range of bacterial species. Sample blanks consisted of unused swabs that were subjected to DNA extraction and confirmed to be DNA amplicon-free. In accordance with the manufacturer’s protocol (QIAGEN), total DNA was eluted in 50 µl of elution buffer, then frozen at -80°C. Finally, total DNA was quantified by Lc-bio Technologies (Hangzhou) Co., Ltd. (Hangzhou, China).

#### 2.3.2 Construction of microbial DNA libraries

Microbial DNA libraries were constructed using the TruSeq Nano DNA LT Library Preparation Kit (FC-121-4001). DNA was fragmented by incubation with dsDNA Fragmentase (NEB, M0348S) at 37°C for 30 min. Library construction begins with fragmented cDNA. Blunt-end DNA fragments were generated using a combination of fill-in reactions and exonuclease activity, then ligated to indexed adapters. Each adapter is designed with a T-base overhang, enabling the seamless ligation of the adapter to the A-tailed fragmented DNA. Next, polymerase chain reaction amplification of the ligated products was conducted under the following conditions: initial denaturation at 95°C for 3 min; 8 cycles of denaturation at 98°C for 15 sec, annealing at 60°C for 15 sec, and extension at 72°C for 30 sec; and a final extension at 72°C for 5 min.

#### 2.3.3 Data processing

The raw sequencing reads underwent processing to extract valid reads for subsequent analysis. Initially, sequencing adapters were eliminated from the reads using cutadapt v1.9. Subsequently, fqtrim v0.94 was employed to trim low-quality reads using a sliding-window algorithm. Finally, the reads were aligned to the host genome using bowtie2 v2.2.0 to eliminate any host contamination. Quality-filtered data were subjected to *de novo* assembly by IDBA-UD v1.1.1 to construct the metagenome for each sample. MetaGeneMark v3.26 was used to predict all coding regions of metagenomic contigs. CD-HIT v4.6.1 was used to remove redundant genes to obtain unigenes. To generate functional information, the unigenes were used as queries for functional database (Kyoto Encyclopedia of Genes and Genomes [KEGG]) analysis by DIAMOND v0.9.14. Based on the above data, as well as the unigene abundance profile, the Mann–Whitney U test, Kruskal–Wallis test (replicated groups), Unweighted Pair Group Method with Arithmetic Mean (UPGMA), and Linear discriminant analysis Effect Size (LEfSe) methods were used to identify features with significant differential abundances across groups. Subsequently, differentially enriched KEGG pathways were identified. Alpha diversity was quantified using the Chao1, good coverage, observed species, and Shannon indices. Beta diversity was calculated using Bray–Curtis distance or Jensen–Shannon Divergence distance.

### 2.4 Metabolomics analysis

#### 2.4.1 Metabolite extraction

Serum and fecal samples (20 µl each) were extracted with 120 µl or 1 ml of precooled 50% methanol, respectively, then incubated at room temperature for 10 min. Subsequently, extraction mixtures were stored at -20°C overnight. After centrifugation (4000 × g for 20 min), supernatants were transferred into 96-well plates and stored at -80°C for liquid chromatography–mass spectrometry (LC-MS) analysis.

#### 2.4.2 LC-MS data acquisition

Samples were analyzed by LC-MS in accordance with the system manufacturer’s instructions. The Vanquish Flex UHPLC system (Thermo Fisher Scientific, Bremen, Germany) was used for chromatographic separation. Reversed phase separation was performed using an ACQUITY UPLC TC column (35°C, 0.4 ml/min, 100 mm × 2.1 mm, 1.8 µm, Waters, Milford, USA); the mobile phase consisted of solvent A (water, 0.1% formic acid) and solvent B (acetonitrile, 0.1% formic acid). The gradient elution conditions were as follows: 0–0.5 min, 5% B; 0.5–7 min, 5% to 100% B; 7–8 min, 100% B; 8–8.1 min, 100% to 5% B; and 8.1–10 min, 5%B. Eluted metabolites were detected using a Q-Exactive high-resolution tandem mass spectrometer (Thermo Fisher Scientific, Bremen, Germany). Precursor spectra (70–1050 m/z) were gathered at 70000 resolutions; the first three configurations for data acquisition used DDA mode. Finally, fragment spectra were collected at 17500 resolutions.

#### 2.4.3 Data processing

The above MS data preprocessing steps (including peak picking, peak grouping, retention time correction, second peak grouping, and annotation of isotopes and adducts) were performed with XCMS software. LC−MS raw data files were converted into mzXML format and then processed by the XCMS, CAMERA and metaX toolbox implemented with the R software. Each peak intensity was recorded; a three-dimensional matrix was generated containing arbitrarily assigned peak indices, sample names, and ion intensity information. The KEGG database and the human metabolome database (HMDB) were used to annotate metabolites. If the mass difference between the observed and the database value was less than 10 ppm, the metabolite was annotated, and the molecular formula of the metabolites was further identified and validated through isotopic distribution measurements. An in-house fragment spectrum library of metabolites was also used to annotate metabolites.

Meta X was used for additional preprocessing of peak intensity data. Features detected in fewer than 50% of quality control samples or 80% of biological samples were removed to improve data quality, the remaining peaks with missing values were imputed using the k-nearest neighbor algorithm, enhancing the overall data quality. Moreover, standard deviations were calculated across quality control samples; samples with standard deviations > 30% were removed. Finally, Student’s t-test was conducted to identify differences in metabolite concentrations. Meta X software was used to distinguish variables across groups. Furthermore, variable importance in projection (VIP) values were calculated; a VIP cut-off value of 1.0 was used to select important features.

### 2.5 Integration of metagenomic with metabolomics data and clinical data

SPSS v26.0 was used to analyze the correlations of highly variable microbiota with blood metabolomics data, fecal metabolomics data, and clinical data. It was also used to evaluate the relationships among metagenomic, metabolomic, and clinical data. *P* values for hypothesis testing were considered statistically significant when *P*<0.05.

## 3 Results

### 3.1 Whole metagenome shotgun sequencing analysis of community structure changes in gut microbiota diversity after SG

**Figure 1:**
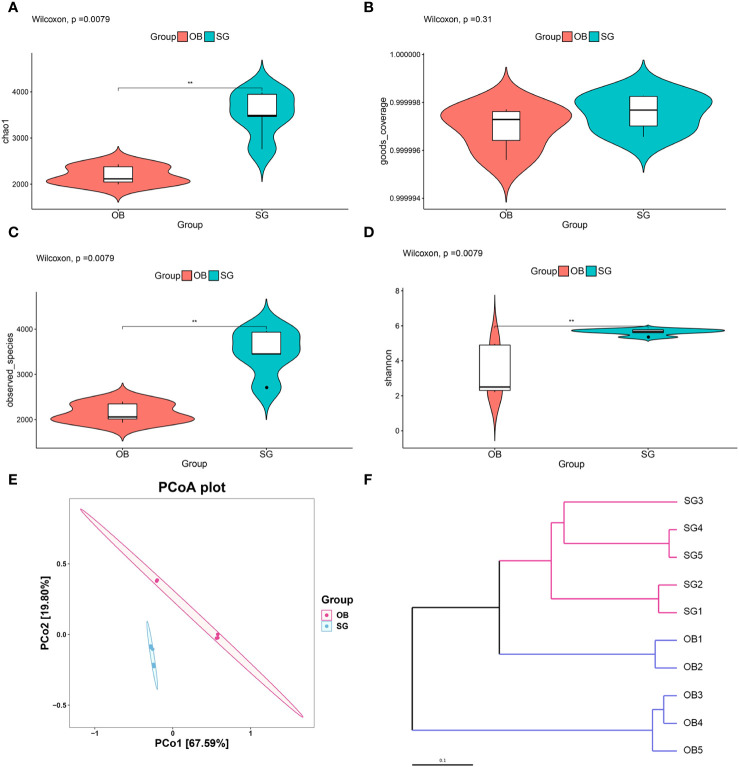
Analysis of community structural changes in the diversity of gut microbiota after sleeve gastrectomy by the whole metageome shotgun sequencing. **(A)** chao1, **(B)** goods coverage, **(C)** observed species and **(D)** Shannon estimators to indicate changes of α-diversity after SG; **(E)** Principal-coordinate analysis (PCoA) of samples derived from two groups based on the Bray-Curtis distance; **(F)** The Unweighted Pair Group Method with Arithmetic Mean (UPGMA) method was used to cluster the samples depending on Bray-Curtis distance. ** means P<0.01.

We collected preoperative and postoperative fecal samples from obese patients who underwent SG; the patients’ clinical characteristics are shown in **Tables 1** and **2**. We defined the preoperative obese patients as the OB group and the post-SG patients as the SG group, the samples and clinical data between these two groups were then analyzed. We first constructed and plotted dilution curves for core and pan genes (**Supplementary Figures 1A, B**). For our sample size, the number of genes was generally stable, indicating that the data were ready for subsequent analysis. Alpha diversity analysis at the species level revealed that the Chao1, observed species, and Shannon indices were statistically significantly higher in the SG group than in the OB group, suggesting that SG caused significantly higher gut microbiota richness, evenness, and diversity (**Figures 1A-D**). We then investigated the effects of SG on gut microbiota composition in obese patients via principal coordinates analysis based on Bray–Curtis distance (**Figure 1E**) and sample clustering using UPGMA (**Figure 1F**). We observed significant differences in microbial composition between the two groups. These results were validated by ANOSIM and Adonis analyses (**Supplementary Figures 1C, D**).

**Table 1:** Demographic characteristics and operation status of study subjects.

Variables	Mean ± SD/n (%)
Participants	5
Age (years)	28.4 ± 3.2
Height (m)	1.7 ± 0.1
Body weight (kg)	127.2 ± 17.1
BMI (kg/m^2^)	43.9 ± 3.8
Gender	
Male	2 (40%)
Female	3 (60%)
Waistline (cm)	133.4 ± 4.7
Hipline (cm)	137.0 ± 7.2
Hospital stays (days)	7.4 ± 1.1
Comorbidities	
Metabolic syndrome	1 (20%)
Hypertension	1 (20%)
Hyperlipemia	1 (20%)
T2DM	1 (20%)
PCOS	1 (20%)
SAHS	5 (100%)
OGTT (mmol/L)	
1 h	10.1 ± 3.8
2 h	8.1 ± 4.1
3 h	5.9 ± 3.4
Operative time (min)	141.0 ± 9.6

Data are expressed as mean ± SD or as numbers and percentages. T2DM, type 2 diabetes mellitus; PCOS, polycystic ovary syndrome; SAHS, sleep apnoea/hypopnoea syndrome; OGTT, oral glucose tolerance tests.

**Table 2:** Clinical parameters before and after laparoscopic sleeve gastrectomy.

Variables	Before LSG	1 month	2 months	3 months
Body weight (kg)	127.2 ± 17.1	108.6 ± 17.1	102.6 ± 16.2*	98.8 ± 16.0*
BMI (kg/m^2^)	43.9 ± 3.8	37.4 ± 3.9*	35.4 ± 3.9*	34.0 ± 3.8*
Alanine aminotransferase (U/L)	52.2 ± 30.3	53.1 ± 29.5	40.5 ± 20.0	22.7 ± 10.8
Aspartate aminotransferase (U/L)	28.8 ± 13.0	28.6 ± 12.8	23.8 ± 7.7	16.6 ± 3.8
Uric acid (μmol/L)	469.0 ± 143.1	538.6 ± 132.0	516.6 ± 132.7	489.2 ± 149.6
Triglyceride (mmol/L)	3.7 ± 5.3	2.4 ± 2.3	1.8 ± 1.4	1.8 ± 0.7
Total cholesterol (mmol/L)	4.7 ± 1.3	4.2 ± 1.4	4.3 ± 1.3	4.9 ± 1.4
High-density lipoprotein (mmol/L)	1.1 ± 0.1	1.2 ± 0.3	1.1 ± 0.3	1.0 ± 0.2
Low-density lipoprotein (mmol/L)	2.3 ± 1.0	2.4 ± 1.0	2.4 ± 1.1	2.4 ± 1.0
Haemoglobin A1c (%)	6.0 ± 1.2	6.1 ± 1.1	5.7 ± 0.5	5.3 ± 0.4
Fasting blood glucose (mmol/L)	5.7 ± 2.8	5.3 ± 1.0	5.1 ± 1.1	5.2 ± 0.9

Data are expressed as mean ± SD. *P < 0.05 versus before LSG. LSG, laparoscopic sleeve gastrectomy; BMI, body mass index.

### 3.2 Changes in gut microbial composition after SG

**Figure 2:**
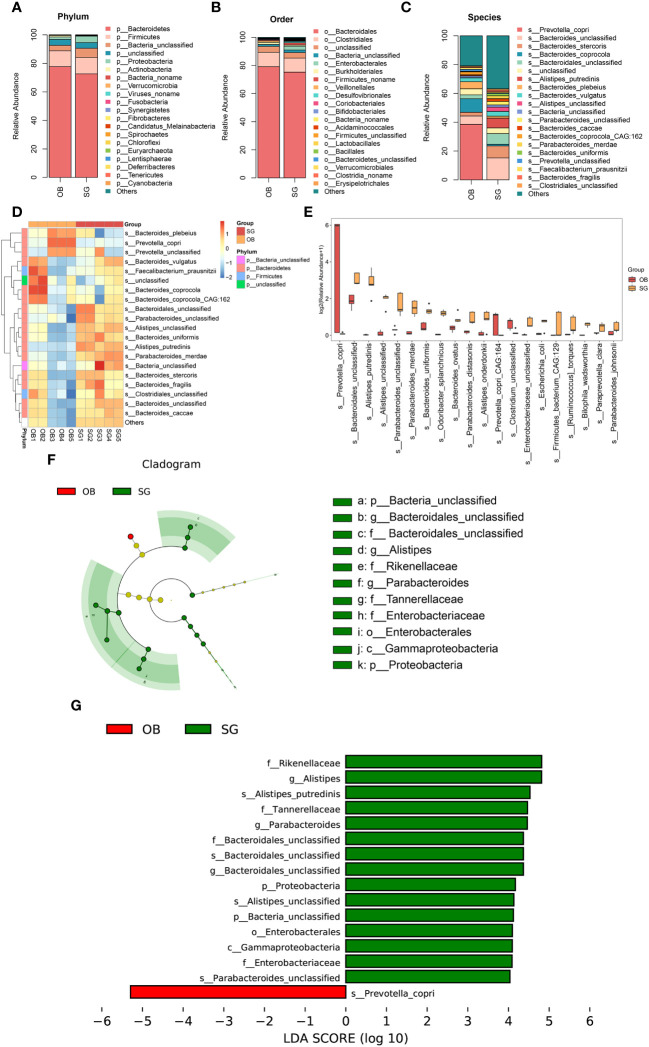
Changes in the gut microbial composition after SG. **(A)** Difference between two groups in the composition of the gut microbiota at the phylum level presented as stacked plot; **(B)** Difference between two groups in the composition of the gut microbiota at the order level presented as stacked plot; **(C)** Difference between two groups in the composition of the gut microbiota at the species presented as stacked plot; **(D)** Changes of post-SG bacterial population at the species level presented as the heat map; **(E)** The TOP 20 differential bacteria species caused by SG, according to the P value; **(F)** Cladogram of LDA Effect Size (LEfSe) for identifying species with significant differences in abundance of the SG group (LDA>4.0); **(G)** LDA score of LDA Effect Size (LEfSe) for identifying species with significant differences in abundance of the SG group (LDA>4.0).

Comparative metagenomics analysis via stacked bar charts and heat maps at the phylum and order level revealed that taxonomic distributions significantly differed between groups (**Figures 2A, B**, **Supplementary Figures 2A–D**). At the phylum level, Verrucomicrobia richness was significantly increased in the SG group, compared with the OB group. Additionally, at the order level, Enterobacterales, Desulfovibrionales, Acidaminococcales, Verrucomicrobiales, and Bacteroidetes were significantly increased after SG, whereas Veillonellales was decreased. To more comprehensively investigate the remodeling effect of SG on gut microbiota, we focused on changes at the species level, which is an advantage of shotgun sequencing and metagenome-wide association studies compared with conventional 16S rDNA sequencing. The resulting data were categorized and presented as stacked bar charts, heat maps, and bar charts (**Figures 2C-E**). At the species level, we found significant increases in Bacteroidales, *Alistipes*, and *Parabacteroides*, along with substantial decreases in *Prevotella* and *Clostridium*, after SG treatment compared with samples collected prior to SG. To identify species with significantly different abundances, we established a linear discriminant analysis (LDA) threshold value of > 3.0 and performed analysis using the LEfSe tool. Numerous taxa were significantly different between the two groups (**Supplementary Figures 2E, F**). To identify biomarkers with greater potential for biological relevance, we established an LDA threshold value of 4.0; using this value, we detected biomarker species for SG, including Rikenellaceae, *Alistipes*, *Parabacteroides*, Bacteroidales, and Enterobacterales; we also detected a biomarker for OB (*Prevotella*) (**Figures 2F, G**).

### 3.3 SG significantly alters the genetic characteristics and functions of gut microbiota

**Figure 3:**
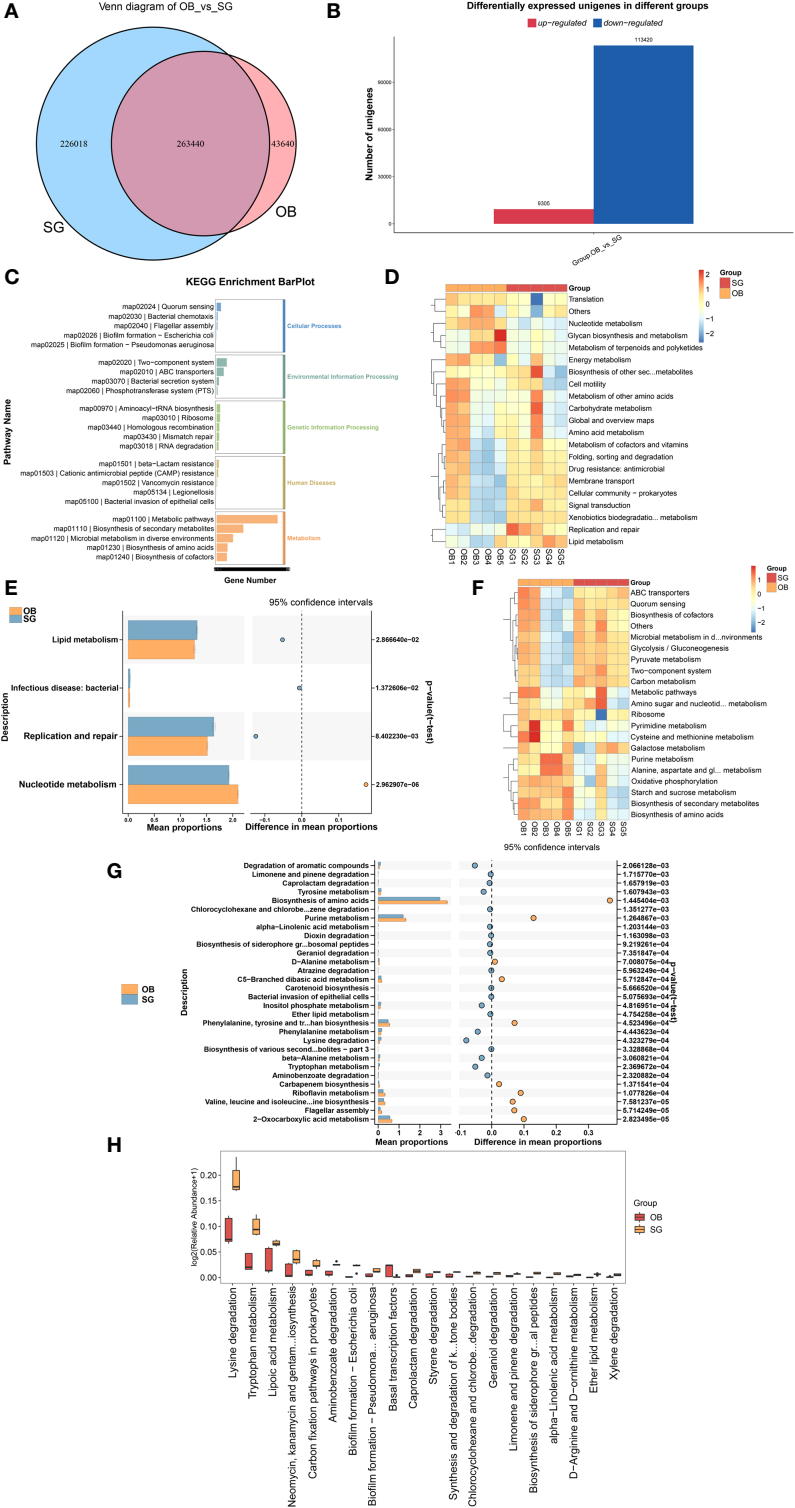
SG significantly alters gut microbiota genetic characteristics and functions **(A)** Venn diagram for differential genes between two groups; **(B)** Altered gut microbial gene counts after SG; **(C)** KEGG enrichment analysis of differential genes between groups; **(D, E)** Altered function of gut microbiota after SG based on KEGG database at the KEGG level 2; **(F, G)** Altered function of gut microbiota after SG based on KEGG database at the KEGG PathwayDefinition level; **(H)** Top 20 most significantly different functions of gut microbiota between the two groups.

Comparative analysis revealed that the number of genes in the gut microbiota was significantly higher in the SG group than in the OB group (**Figure 3A**). Furthermore, compared with the OB group, the SG group displayed 113,420 upregulated genes and 9,305 downregulated genes (**Figure 3B**). Analysis using the KEGG database revealed that the differential genes were mainly enriched in metabolism-related pathways, suggesting that the changes in gut microbiota after SG are associated with improvements in host metabolic status (**Figure 3C**, **Supplementary Figure 3A**). Next, we analyzed differences in microbial function between the two groups based on KEGG database findings. We found no statistically significant differences between the two groups at KEGG level1 (**Supplementary Figure 3B**). However, a significant difference between the two groups was present at KEGG level2 (**Figure 3D**, **Supplementary Figures 3C–E**). Among the differential pathways, the lipid metabolism pathway was statistically significantly enhanced in the SG group (**Figure 3E**). Further analysis according to KEGG pathway definitions revealed additional differences in metabolic pathways (**Figure 3F**, **Supplementary Figures 3F–H**). We identified the top 30 functional pathways with the greatest differences in KEGG PathwayDefinition (**Figure 3G**), then ranked the top 20 and depicted them in a box plot (**Figure 3H**). The results showed that the levels of amino acid metabolism (e.g., lysine and tryptophan) and lipid metabolism (e.g., fatty acids, ether lipids, and α-linolenic acid) pathway components were significantly increased after SG.

### 3.4 Altered gut metabolite profiles after SG

**Figure 4:**
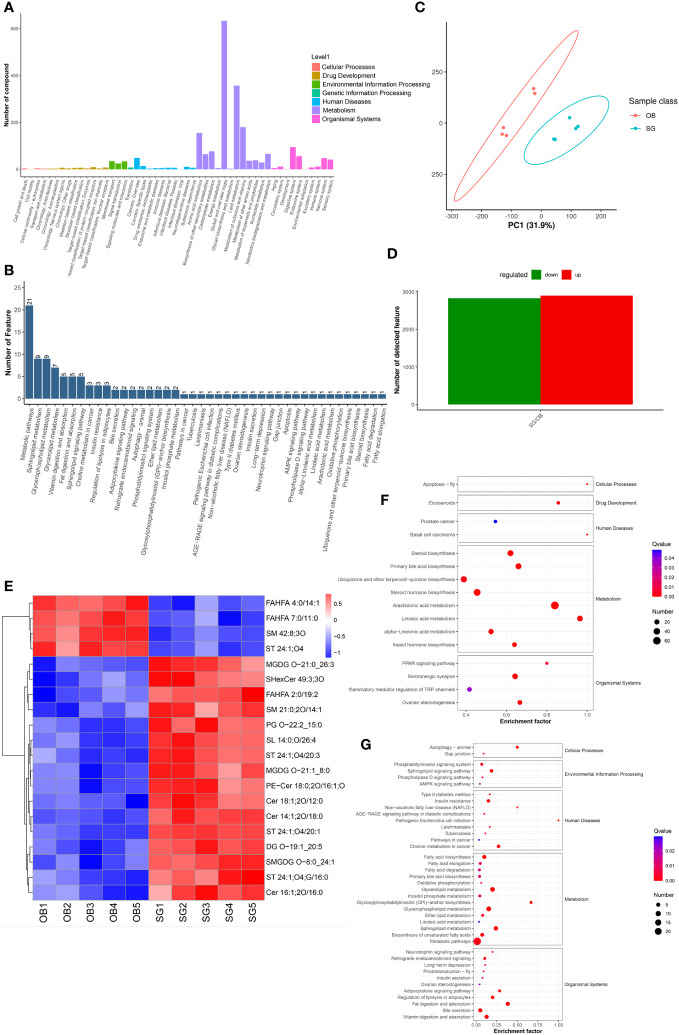
Comparative metabolomics analysis revealed the alteration in gut metabolites after SG. **(A)** Annotation of overall gut metabolic features identified by MS1 based on the KEGG database; **(B)** Annotation of overall gut metabolic features identified by MS2 based on the KEGG database; **(C)** PCA for gut metabolites from two groups; **(D)** Statistical bar graph of the change in the count of differentially metabolized ions, caused by SG; **(E)** Heat maps revealing the different intestinal metabolites between the two groups; **(F)** The KEGG Enrichment Analysis for gut metabolites identified by MS1; **(G)** The KEGG Enrichment Analysis for gut metabolites identified by MS2.

Non-target metabolome sequencing of fecal samples yielded 10955 features in negative ion mode (NIM) and 18498 features in positive ion mode (PIM), with 1194 and 986 ions of all secondary metabolites identified, respectively. And there were 5576 and 9053 annotated primary metabolites in NIM and PIM, respectively (**Table 3**). In single-stage mass spectrometry (MS1) analysis, the data identified were applied as a bulk query to the human metabolome database (HMDB) and annotated with 4333 and 7296 individual samples of the identifying features identified in the PIM and NIM, respectively, and the highest abundant metabolites were lipids or lipid analogues (**Table 3**; **Supplementary Figure 4A**). It was found that they were mainly enriched in metabolic functional pathways, including lipid metabolism, amino acid metabolism, cofactors, and vitamins, and were also closely associated with endocrine disorders, in addition to digestive disease, as shown by MS1 analysis based on the KEGG (**Figure 4A**). Afterwads, we performed secondary-mass spectrometry analysis (MS2) and found that the metabolites were mainly related to lipid metabolism such as sphingolipids, glycerophospholipids, and unsaturated fatty acids (**Figure 4B** and **Supplementary Figure 4B**). Subsequently, PCA and PLSDA showed significant group differences between the SG and OB groups (**Figure 4C** and **Supplementary Figure 4C**). There were 2899 metabolic ions upregulated and 2827 metabolic ions downregulated in the SG group compared to the OB group (**Figure 4D**). The difference in metabolites between the two groups was visualized by the volcano and heat maps (**Supplementary Figure 4D** and **4E**). We then listed the 20 metabolites identified by MS2 as differential features with the highest differences according to P values by annotating them into the database (**Figure 4E**), and demonstrated a significant increase in small molecular lipid molecules including free fatty acids, triglycerides, ceramides, etc., and an increase in esterified bile acids after SG, predominantly esterified deoxycholic acid and esterified glyodeoxycholic acid. In order to clarify the functions of the metabolites, we annotated them into the KEGG database according to the identified primary and secondary metabolites. The different primary metabolites between the two groups were mainly enriched in unsaturated fatty acid (PUFAs) metabolic pathways such as linolenic acid, linoleic acid, and arachidonic acid and were closely associated with metabolic pathways such as primary bile acid synthesis and steroid synthesis (**Figure 4F**). Secondary metabolites are likewise mainly enriched in metabolism-related pathways, including fatty acid synthesis, prolongation, degradation, glycerophospholipids, ether lipids, linoleic acid, etc. (**Figure 4G**). All the above results can be corresponded to metagenome-wide association studies above.

**Table 3:** Identification of the fecal metabolome.

Mode	All	MS2	HMDB	KEGG	Annotated
Negative	10955	1194	4333	2371	5576
Positive	18498	986	7926	4268	9053

### 3.5 Serum metabolic profiles change after SG

**Figure 5:**
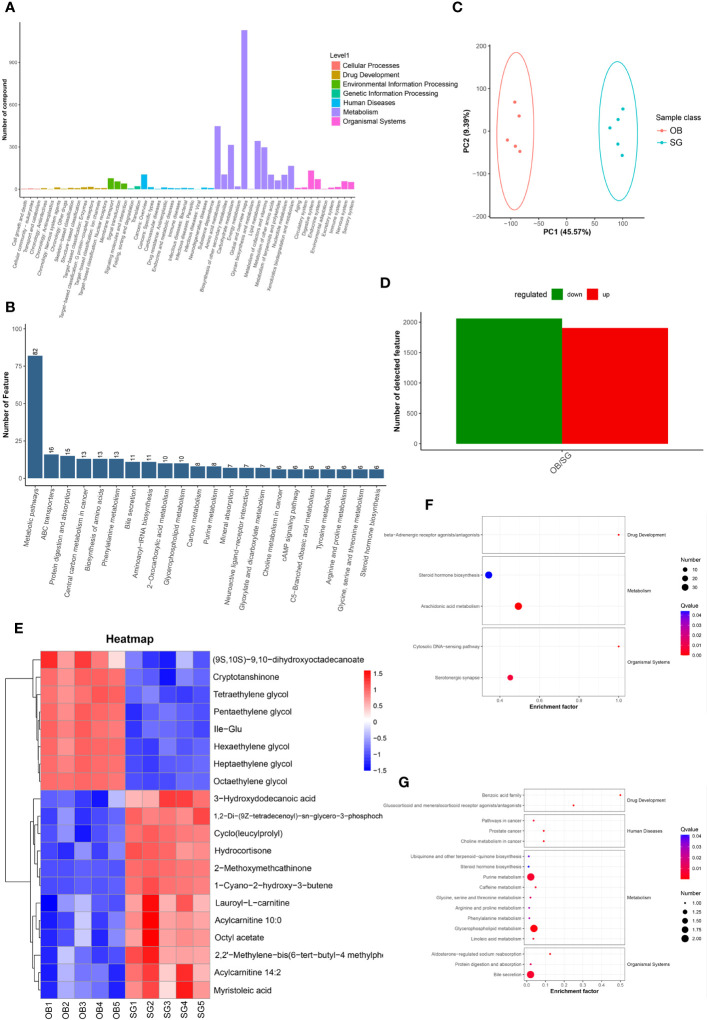
Comparative metabolomics analysis revealed the alteration in serum metabolites after SG. **(A)** Annotation of overall serum metabolic features identified by MS1 based on the KEGG database; **(B)** Annotation of overall serum metabolic features identified by MS2 based on the KEGG database; **(C)** PCA for serum metabolites from two groups; **(D)** Statistical bar graph of the change in the count of differentially metabolized ions, caused by SG; **(E)** Heat maps revealing the different serum metabolites between the two groups; **(F)** The KEGG Enrichment Analysis for serum metabolites identified by MS1; **(G)** The KEGG Enrichment Analysis for serum metabolites identified by MS2.

Non-targeted metabolomic profiling of serum samples revealed 11451 and 15496 metabolic features according to MS1, 348 and 477 metabolic features according to MS2, and 6062 and 7812 annotated primary metabolites in NIM and PIM, respectively (**Table 4**). Similar to the fecal metabolomic analysis, the primary metabolites identified were mainly lipids or lipid analogs, according to the HMDB (**Supplementary Figure 5A**). Meanwhile, on the basis of the KEGG database, the functional pathways of the primary metabolites were amino acid metabolism, lipid metabolism, cofactor and vitamin metabolism, which was consistent with the results obtained from the fecal samples (**Figure 5A**). We further annotated the features obtained from secondary mass spectrometry into KEGG, showing that the functions of the metabolites in the serum samples are mainly closely related to the metabolic pathways (**Figure 5B** and **Supplementary Figure 5B**). We identified metabolites that differed between the SG and OB groups, then analyzed those metabolic features via PCA (**Figure 5C**) and PLSDA (**Supplementary Figure 5C**); we found that samples demonstrated similarity within the OB and SG groups, whereas they significantly differed between groups. After SG, 2060 features were upregulated, and 1913 features were downregulated (**Figure 5D**). Metabolites with changes after SG were visualized using heat and volcano plots (**Supplementary Figures 5D, E**). To further clarify the differential metabolites between groups, the identified metabolite ions were annotated into the database and a visual analysis of the TOP20 differential metabolites was performed based on *P* values (**Figure 5E**). The levels of dihydroxyoctadecanoate, cryptotanshinone, tetraethylene glycol, and isoleucine- glutamate (Ile-Glu) significantly decreased after SG, whereas the levels of 3-hydroxydodecanoic acid, hydrocortisone, lauroyl-L-carnitine, and acylcarnitine significantly increased. Finally, we performed separate functional analyses of primary and secondary differential metabolites via KEGG enrichment analysis and bubble mapping. Differential primary metabolites were mainly associated with linoleic acid, a type of polyunsaturated fatty acids (PUFAs), and steroid hormone biosynthetic pathways (**Figure 5F**). In contrast, differential secondary metabolites were mainly associated with purine metabolism, glycerophosphate metabolism, and linoleic acid metabolism (**Figure 5G**).

**Table 4:** Identification of the serum metabolome.

Mode	All	MS2	HMDB	KEGG	Annotated
Negative	11451	348	5065	4431	6062
Positive	14296	477	6815	5523	7812

### 3.6 Combined multi-omics analysis reveals SG-mediated remodeling of the gut microenvironment

**Figure 6:**
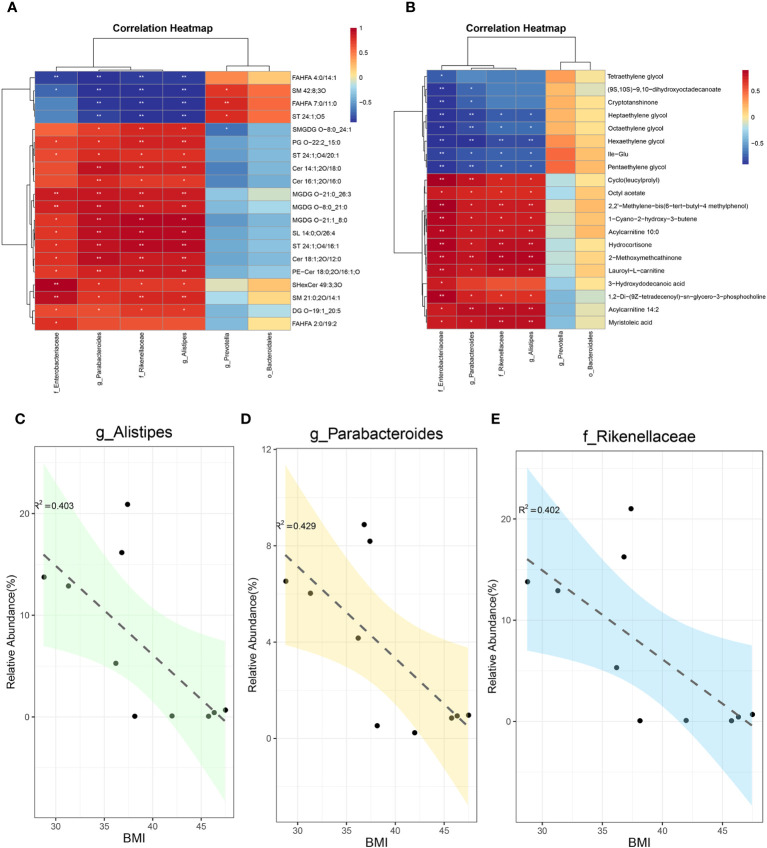
Integreted multi-omics analysis revealed the remodeling of intestinal microenvironment mediated by SG. **(A)** The correlation analysis between specific differential bacteria and TOP 20 fecal differential metabolites; **(B)** The correlation analysis between specific differential bacteria and TOP 20 serum differential metabolites; **(C)** The linear correlation analysis between abundance of Alistipes and BMI; **(D)** The linear correlation analysis between abundance of Parabacteroides and BMI; **(E)** The linear correlation analysis between abundance of Rikenellaceae and BMI.

For systematic assessment of the mechanism by which SG alleviates obesity through gut microenvironment remodeling, we performed integrated analysis using a multi-omics approach in combination with clinical data. During whole metagenome shotgun sequencing and LEfSe analysis, we tentatively identified several microbes with substantial changes in abundance after SG, including Rikenellaceae, *Alistipes*, *Parabacteroides*, Bacteroidales, Enterobacterales, and *Prevotella*; we compared them with differential metabolites from fecal samples (**Supplementary Figure 6A**) and serum samples (**Supplementary Figure 6B**). Because of the large amount of data, we performed correlation analysis between the above differential microbiota and the 20 most significantly different metabolites in feces (**Figure 6A**) and serum (**Figure 6B**). We found that Rikenellaceae, *Alistipes*, *Parabacteroides*, and Enterobacterales had statistically significant correlations with most of the differential metabolites, whereas *Prevotella* and Bacteroidales were poorly correlated with differential metabolites; these findings were consistent across metabolites from fecal and serum samples, suggesting that the first four bacteria contribute to the weight-loss effect of SG. Accordingly, we performed a linear analysis of BMI, an important clinical indicator of obesity, using the above six groups of bacteria. We found that Rikenellaceae, *Alistipes*, and *Parabacteroides* were correlated with BMI (**Figures 6C-E**), whereas the other three microbiota were not correlated with BMI (**Supplementary Figures 6C–E**). Taken together, these results suggest that the increased abundances of Rikenellaceae, *Alistipes*, and *Parabacteroides* in the gut microbiota after SG have important effects on the outcome of SG-mediated weight reduction therapy.

## 4 Discussion

Obesity and its related complications pose a serious public health threat worldwide (1, 24). In the context of the COVID-19 pandemic, the metabolic disease aspect of obesity has received increasing attention based on findings that it increases COVID-19 risk and severity (25, 26). SG as a common bariatric surgery operation, has demonstrated a substantial weight-loss effect in obese patients; it also alleviates obesity-related complications and delays their progression (27, 28). However, additional research is needed to fully elucidate the therapeutic mechanisms of SG, particularly from an gut microecological perspective. In the present study, we utilized a multi-omics approach to analyze changes in gut microbiota and metabolites after SG among obese patients from Shandong, China. Such changes had important effects on host metabolism and might offer biomarkers forgut microecology after SG.

Previous studies have shown that bariatric surgery can significantly contribute to improvement in the gut dysbacteriosis associated with obesity (29). SG alters the normal structure of the gastrointestinal tract and rearranges the gut microecology whcih may play a central role in the effect of SG. In this study, the whole metagenome shotgun sequencing revealed a significant increase in alpha diversity after SG, suggesting significant increases in microbiota richness and diversity. Subsequently, principal coordinates analysis of samples from the OB and SG groups revealed similarities within each group and differences between groups. Based on these results, we conducted further exploration of the gut microbiota composition. LEfSe analysis was performed to investigate the effect of SG on gut microbial community composition at various levels, especially the species level. Rikenellaceae, *Alistipes*, *Parabacteroides*, Bacteroidales, and Enterobacterales were identified as characteristic bacteria in the SG group; *Prevotella* was identified as the characteristic bacteria in the OB group. In a previous study, Rikenellaceae abundance was considerably decreased in the intestines of obese patients compared with lean patients; its abundance was positively correlated with the level of ClpB, a bacterial protein that was negatively correlated with BMI, waist circumference, and total fat mass (30). *Alistipes* abundance in the gut microbiota is significantly dysregulated in obese mice; modifications that restore its abundance can partially reverse lipid metabolism disorders (31). The abundance of *Alistipes* gradually decreases as NAFLD progresses to nonalcoholic steatohepatitis and cirrhosis (32). *Parabacteroides* spp. are suspected to mediate anti-obesity effects through enhancement of fat thermogenesis, maintenance of gut integrity, and reduction of the serum insulin resistance level (33). Fecal microbiota transplantation from bariatric surgery patients to experimental mice revealed a significant increase in *Parabacteroides* abundance in the mice, along with improved glucose metabolism (34). Similarly, Bacteroidales spp. are closely associated with obesity-related diseases such as NAFLD and hepatic fibrosis. Additionally, they have demonstrated significant correlations with the abundances of bile acids, such as deoxycholic acid (35) Enterobacterales spp. are generally the focus of infectious disease research, but their roles in lipid metabolism and related fields have not been extensively explored (36). Considering the increased abundance of Enterobacterales observed after SG in the present study, the roles of Enterobacterales spp. in metabolism require further investigation. Notably, the abundance of *Prevotella* in the intestinal contents of rats was significantly increased after SG compared with the control group, indicating that *Prevotella* spp. could have a therapeutic effect on the disturbance of host glucose metabolism by increasing bile acid production and activating farnesoid X receptor (FXR) (37). However, our findings suggested that *Prevotella* abundance decreased after SG and this discrepancy may be the result of species differences, sample sources, differences in dietary structure, and differences in study location. Furthermore, this difference illustrates the need for analyses of gut microbiology to be conducted via clinical trials, rather than animal models alone.

For the alteration of gut microbial function after SG, a series of related analyses at the gene level were performed and showed that a number of genes in the gut contents was substantially increased after SG. Subsequently, we elucidated the mechanism by which SG improves obesity through the gut microbiota, using an approach that involved KEGG-mediated functional annotation of the microbiota at various levels. We found that overall metabolic functions (e.g., fatty acids, amino acids such as tryptophan, and PUFAs metabolic pathways) were generally upregulated after SG. Tryptophan metabolism plays an essential role in the microbiota–gut–brain axis; it is also involved in many psychiatric and neurological disorders (38). Moreover, tryptophan-related metabolites in the gut environment can bind to aryl hydrocarbon receptor ligands and regulate metabolic functions (39). In patients with metabolic disorders, an important gut finding is a decrease in tryptophan, which leads to a decrease in aryl hydrocarbon receptor binding and the activation of both GLP-1 and interleukin-22; this pathway ultimately promotes inflammation, insulin resistance, and hepatic steatosis (40, 41). PUFAs are susceptible to oxidative degradation because of their unstable double bonds; this degradation can lead to tissue damage (42). However, the gut microbiota can allow the host to resist obesity during high-fat diet intake via regulation of PUFAs metabolism (43). The present study also revealed that SG significantly increased the levels of tryptophan and PUFAs, helping to explain the mechanism underlying the superior weight-loss effect of SG from the perspective of gut microecology.

According to the whole metagenome shotgun sequencing, SG could lead to changes in the metabolic function of gut microbiota. Therefore, we conducted non-targeted metabolomic profiling of fecal samples from obese patients undergoing SG; this approach enabled the identification of a greater number of small molecule metabolites. Metabolic ions identified in fecal samples were mainly lipid molecules, which may be associated with the production of short-chain fatty acids by the gut microbiota that were subsequently absorbed by the intestine and finally excreted into feces (44); our whole metagenome shotgun sequencing results corroborated this previous finding. Furthermore, we observed a small increase in the number of gut metabolites after SG, which may be associated with greater gut microbial diversity and richness. We focused on the top 20 different small molecule metabolites according to *P*-values. A decrease in sphingomyelin in the gut environment was observed after SG. Sphingomyelin has been significantly positively correlated with the level of serum cholesterol; a lower level of sphingomyelin was significantly associated with alleviation of hypercholesterolemia (45). We also found that fatty acid esters of hydroxy fatty acids (FAHFAs) were significantly downregulated after SG. Notably, the effects of FAHFAs on metabolism are controversial; some studies show anti-inflammatory and anti-diabetic effects (46), whereas other studies show contrasting findings (47). Thus, the roles of FAHFAs in metabolism, particularly lipid metabolism, require further investigation. Additionally, we found that SG led to increases in gut sterol sulfate, glycodeoxycholic acid, and phosphatidylglycerol. In a previous study, sterol sulfate tended to be negatively correlated with serum cholesterol and low-density lipoprotein (48). Glycodeoxycholic acid was negatively correlated with insulin clearance, and patients with obesity generally exhibited lower insulin clearance (49). Phosphatidylglycerol remodeling can significantly improve hepatic steatosis by stabilizing mitochondrial structure (50). Although SG leads to a significant weight-loss effect and improves lipid metabolism, we observed significant increases in the levels of free fatty acids and triglycerides in fecal samples collected from patientsunderwent SG. Recent studies have shown that short-chain fatty acids, among the major products of gut microbiota, can function as signaling molecules to regulate lipid metabolism and glucose homeostasis in liver, adipose tissue, and skeletal muscle, thereby exerting anti-obesity effects (51, 52). In summary, the increased levels of various metabolites with favorable roles in the regulation of glucolipid metabolism after SG, along with decreases in the levels of some metabolically harmful substances, provide important insights concerning the weight-loss therapeutic effect of SG from an gut metabolite perspective.

Considering the altered metabolic profile observed after SG, we profiled serum small molecule metabolites. We initially analyzed the metabolic ions identified in our study which were dominated by lipid-like molecules and closely associated with metabolism-related functional pathways, such as amino acids and lipids. Analysis of differential metabolites after SG revealed a significant reduction in isoleucine, a type of branched-chain amino acid; this finding was consistent with the results of a study regarding altered serum metabolic profiles after bariatric surgery (53). Moreover, it may partially explain the mechanism of SG because an elevated serum branched-chain amino acid level was strongly associated with obesity, insulin resistance, and T2DM in humans (54). Additionally, hexaethylene glycol, heptaethylene glycol, cryptotanshinone, and tetraethylene glycol were substantially lower after SG. To our knowledge, the roles of these four metabolites in glucose–lipid metabolism and obesity have not been determined thus far; follow-up studies are needed to explore whether they can be used as therapeutic targets for obesity. Furthermore, we found increases in serum myristoleic acid, hydrocortisone, lauroyl-L-carnitine, and acylcarnitine after SG, compared with samples collected before SG. An increase in myristoleic acid, a class of long-chain fatty acids, has potential anti-obesity effects that involve activating brown adipose tissue and promoting the production of beige fat. In a previous study, the serum hydrocortisone concentration reportedly was decreased after SG (55), which is inconsistent with our findings. This discrepancy is probably related to regional differences in dietary structure, which may influence bacterial metabolites (56). An elevated level of acylcarnitine may enhance mitochondrial β-oxidation, thereby reducing lipid accumulation (57). However, lauroyl-L-carnitine had a positive association with T2DM in a clinical study (58).

The human metabolic profile involves multidimensional crosstalk, and a single omics approach cannot fully illuminate the intricate mechanisms by which SG improves metabolism. Therefore, a multi-omics approach was performed in combination with an analysis of clinical data. We initially identified several candidate bacterial groups via LEfSe analysis, then explored their relationships with differential metabolites in feces and serum samples. We identified four types of bacteria—Rikenellaceae, *Alistipes*, *Parabacteroides*, and Enterobacterales—that were negatively correlated with pro-obesity metabolites (e.g., sphingomyelin) and positively correlated with anti-obesity metabolites (e.g., myristoleic acid). Additionally, linear correlation analysis between these bacteria and BMI revealed that only Rikenellaceae, *Alistipes*, and *Parabacteroides* were both significantly associated with differential metabolites and linearly correlated with BMI. Thus, we concluded that Rikenellaceae, *Alistipes*, and *Parabacteroides* are key bacteria with important roles in the ability of SG to alleviate obesity.Among these, Alistipes was identified as a novel distinctive feature in gut microbiota after SG in this study, compared to previous studies using 16S ribosomal RNA sequencing to detect the effects of SG on gut microbiota (22). These bacteria represent potential targets for future obesity therapies, and set the stage for exploring new non-invasive modality in treating obesity and its related complications.

However, there are several limitations to our study. Due to the fact that many patients undergoing bariatric surgery come from other regions, it is challenging for them to return to our hospital for follow-up examinations due to the long distances involved. Additionally, post-surgery constipation experienced by many patients makes it difficult to collect fecal samples. Consequently, the sample size in this study is relatively small. A larger sample cohort experiment is still needed in the future to further verify the above conclusion, and provide novel targeted drugs with specific gut microbiota for obese patients. Moreover, there was only a correlation between these bacteria we identified and the altered metabolic and clinical parameters. In order to establish causation, additional animal experiments should be further performed.

## 5 Conclusions

In the present study, we collected fecal and serum samples from obese patients before and after SG, and then classified and functionally analyzed the samples using the whole metagenome shotgun sequencing and the non-targeted metabolomic profiling. We revealed the remodeling of gut microbiota by SG through a multi-omics approach, and identified Rikenellaceae, *Alistipes*, and *Parabacteroide* as the key strains of SG exerting weight-loss effects. This study elucidated the mechanism of SG from the perspectives of gut microbiota and metabolites.

## Trial registration

This study was granted approval by the Ethics Review Committee of the First Affiliated Hospital of Shandong First Medical University (Permission number: 2023-S358). Registered 12 May 2023. Retrospectively registered.

## Ethics approval and consent to participate

This study was granted approval by the Ethics Review Committee of the First Affiliated Hospital of Shandong First Medical University (Permission number: 2023-S358).

## Permission to reuse and copyright

Permission must be obtained for use of copyrighted material from other sources (including the web). Please note that it is compulsory to follow figure instructions.

## Data availability statement

The datasets presented in this study can be found in online repositories. The names of the repository/repositories and accession number(s) can be found in the article/https://doi.org/10.6084/m9.figshare.23659710.

## Ethics statement

The studies involving humans were approved by Ethics Review Committee of the First Affiliated Hospital of Shandong First Medical University. The studies were conducted in accordance with the local legislation and institutional requirements. The participants provided their written informed consent to participate in this study.

## Author contributions

CL: Formal Analysis, Investigation, Writing – original draft. QX: Formal Analysis, Investigation, Writing – original draft. SD: Conceptualization, Writing – review & editing. HD: Investigation, Validation, Writing – original draft. BL: Investigation, Writing – original draft. DZ: Writing – original draft. YL: Writing – original draft. LL: Data curation, Writing – original draft. QL: Writing – original draft. YuC: Supervision, Writing – original draft. JW: Formal Analysis, Validation, Writing – review & editing. JZ: Project administration, Validation, Writing – review & editing. MZ: Project administration, Supervision, Validation, Writing – review & editing. YiC: Methodology, Project administration, Supervision, Validation, Writing – review & editing. GZ: Conceptualization, Funding acquisition, Project administration, Validation, Writing – review & editing.

## Supplementary material

Supplementary Figure 1 **(A)** The dilution curves for Core genes; **(B)** The dilution curves for Pan genes; **(C)** The result for the Analysis of similarities (Anosim) between the two groups; **(D)** The result for the Adonis analysis between the two groups.

Supplementary Figure 2 **(A)** Changes of post-SG bacterial population at the phylum level presented as the heat map; **(B)** Changes of post-SG bacterial population at the order level presented as the heat map; **(C)** The TOP 20 differential microbial composition at the phylum level caused by SG, according to the P value; **(D)** The TOP 20 differential microbial composition at the order level caused by SG, according to the P value; **(F)** Cladogram of LDA Effect Size (LEfSe) for identifying species with significant differences in abundance of the SG group (LDA>3.0); **(G)** LDA score of LDA Effect Size (LEfSe) for identifying species with significant differences in abundance of the SG group (LDA>3.0).

Supplementary Figure 3 **(A)** The KEGG enrichment analysis of differential genes between groups presented as the scatterplot map; **(B)** Altered function of gut microbiota after SG based on KEGG database at the level1; **(C, D, E)** Altered function of gut microbiota after SG based on KEGG database at the KEGG level2; **(F, G, H)** Altered function of gut microbiota after SG based on KEGG database at the KEGG PathwayDefinition level.

Supplementary Figure 4 **(A)** the HMDB super class graph of the overall annotated gut metabolic features identified by MS1 based on the HMDB database; **(B)** the scatterplot graph of the overall annotated gut metabolic features identified by MS2 based on the KEGG database; **(C)** The partial least squares discriminant analysis (PLSDA) for gut metabolites between the two groups; **(D)** The volcano plot revealing the different gut metabolites between the two groups; **(E)** The heat maps revealing the different gut metabolites between the two groups.

Supplementary Figure 5 **(A)** the HMDB super class graph of the overall annotated serum metabolic features identified by MS1 based on the HMDB database; **(B)** the scatterplot graph of the overall annotated serum metabolic features identified by MS2 based on the KEGG database; **(C)** The partial least squares discriminant analysis (PLSDA) for serum metabolites between the two groups; **(D)** The volcano plot revealing the different serum metabolites between the two groups; **(E)** The heat maps revealing the different serum metabolites between the two groups.

Supplementary Figure 6 **(A)** The correlation analysis between differential gut microbiota and gut differential metabolites; **(B)** The correlation analysis between differential gut microbiota and serum differential metabolites; **(C)** The linear correlation analysis between abundance of Bacteroidales and BMI; **(D)** The linear correlation analysis between abundance of Enterobacteriaceae and BMI; **(E)** The linear correlation analysis between abundance of Prevotella and BMI.

## References

[1] Blüher M. Obesity: global epidemiology and pathogenesis. Nat Rev Endocrinol (2019) 15(5):288–98. doi: 10.1038/s41574-019-0176-8 30814686

[2] Wang L Zhou B Zhao Z Yang L Zhang M Jiang Y. Body-mass index and obesity in urban and rural China: findings from consecutive nationally representative surveys during 2004-18. Lancet (2021) 398(10294):53–63. doi: 10.1016/s0140-6736(21)00798-4 34217401 PMC7617101

[3] Fan Y Pedersen O. Gut microbiota in human metabolic health and disease. Nat Rev Microbiol (2021) 19(1):55–71. doi: 10.1038/s41579-020-0433-9 32887946

[4] McAllister EJ Dhurandhar NV Keith SW Aronne LJ Barger J Baskin M. Ten putative contributors to the obesity epidemic. Crit Rev Food Sci Nutr (2009) 49(10):868–913. doi: 10.1080/10408390903372599 19960394 PMC2932668

[5] Lindell AE Zimmermann-Kogadeeva M Patil KR. Multimodal interactions of drugs, natural compounds and pollutants with the gut microbiota. Nat Rev Microbiol (2022) 20(7):431–43. doi: 10.1038/s41579-022-00681-5 PMC7615390 35102308

[6] Li J Jia H Cai X Zhong H Feng Q Sunagawa S. An integrated catalog of reference genes in the human gut microbiome. Nat Biotechnol (2014) 32(8):834–41. doi: 10.1038/nbt.2942 24997786

[7] Aron-Wisnewsky J Warmbrunn MV Nieuwdorp M Clément K. Metabolism and metabolic disorders and the microbiome: the intestinal microbiota associated with obesity, lipid metabolism, and metabolic health-pathophysiology and therapeutic strategies. Gastroenterology (2021) 160(2):573–99. doi: 10.1053/j.gastro.2020.10.057 33253685

[8] Li R Huang X Liang X Su M Lai KP Chen J. Integrated omics analysis reveals the alteration of gut microbe-metabolites in obese adults. Brief Bioinform (2021) 22(3):bbaa165. doi: 10.1093/bib/bbaa165 32770198

[9] Cox LM Blaser MJ. Antibiotics in early life and obesity. Nat Rev Endocrinol (2015) 11(3):182–90. doi: 10.1038/nrendo.2014.210 PMC4487629 25488483

[10] de Vos WM Tilg H Van Hul M Cani PD. Gut microbiome and health: mechanistic insights. Gut (2022) 71(5):1020–32. doi: 10.1136/gutjnl-2021-326789 PMC8995832 35105664

[11] Le Roy T Moens de Hase E Van Hul M Paquot A Pelicaen R Régnier M. Dysosmobacter welbionis is a newly isolated human commensal bacterium preventing diet-induced obesity and metabolic disorders in mice. Gut (2022) 71(3):534–43. doi: 10.1136/gutjnl-2020-323778 PMC8862106 34108237

[12] den Besten G Bleeker A Gerding A van Eunen K Havinga R van Dijk TH. Short-chain fatty acids protect against high-fat diet-induced obesity via a PPARγ-dependent switch from lipogenesis to fat oxidation. Diabetes (2015) 64(7):2398–408. doi: 10.2337/db14-1213 25695945

[13] Frost G Sleeth ML Sahuri-Arisoylu M Lizarbe B Cerdan S Brody L. The short-chain fatty acid acetate reduces appetite via a central homeostatic mechanism. Nat Commun (2014) 5:3611. doi: 10.1038/ncomms4611 24781306 PMC4015327

[14] Jia W Xie G Jia W. Bile acid-microbiota crosstalk in gastrointestinal inflammation and carcinogenesis. Nat Rev Gastroenterol Hepatol (2018) 15(2):111–28. doi: 10.1038/nrgastro.2017.119 PMC5899973 29018272

[15] Miras AD le Roux CW. Metabolic surgery: shifting the focus from glycaemia and weight to end-organ health. Lancet Diabetes Endocrinol (2014) 2(2):141–51. doi: 10.1016/s2213-8587(13)70158-x 24622718

[16] Nguyen NT Varela JE. Bariatric surgery for obesity and metabolic disorders: state of the art. Nat Rev Gastroenterol Hepatol (2017) 14(3):160–9. doi: 10.1038/nrgastro.2016.170 27899816

[17] Brajcich BC Hungness ES. Sleeve gastrectomy. Jama (2020) 324(9):908. doi: 10.1001/jama.2020.14775 32870299

[18] Aminian A. Sleeve gastrectomy: metabolic surgical procedure of choice? Trends Endocrinol Metab (2018) 29(8):531–4. doi: 10.1016/j.tem.2018.04.011 29804898

[19] Miras AD le Roux CW. Mechanisms underlying weight loss after bariatric surgery. Nat Rev Gastroenterol Hepatol (2013) 10(10):575–84. doi: 10.1038/nrgastro.2013.119 23835488

[20] Steenackers N Vanuytsel T Augustijns P Tack J Mertens A Lannoo M. Adaptations in gastrointestinal physiology after sleeve gastrectomy and Roux-en-Y gastric bypass. Lancet Gastroenterol Hepatol (2021) 6(3):225–37. doi: 10.1016/s2468-1253(20)30302-2 33581761

[21] Liu R Hong J Xu X Feng Q Zhang D Gu Y. Gut microbiome and serum metabolome alterations in obesity and after weight-loss intervention. Nat Med (2017) 23(7):859–68. doi: 10.1038/nm.4358 28628112

[22] Ikeda T Aida M Yoshida Y Matsumoto S Tanaka M Nakayama J. Alteration in faecal bile acids, gut microbial composition and diversity after laparoscopic sleeve gastrectomy. Br J Surg (2020) 107(12):1673–85. doi: 10.1002/bjs.11654 32432347

[23] Le Chatelier E Nielsen T Qin J Prifti E Hildebrand F Falony G. Richness of human gut microbiome correlates with metabolic markers. Nature (2013) 500(7464):541–6. doi: 10.1038/nature12506 23985870

[24] American Diabetes Association Professional Practice Committee. 8. Obesity and weight management for the prevention and treatment of type 2 diabetes: standards of medical care in diabetes-2022. Diabetes Care (2022) 45(Suppl 1):S113–s124. doi: 10.2337/dc22-S008 34964843

[25] Stefan N Birkenfeld AL Schulze MB. Global pandemics interconnected - obesity, impaired metabolic health and COVID-19. Nat Rev Endocrinol (2021) 17(3):135–49. doi: 10.1038/s41574-020-00462-1 33479538

[26] Le Brocq S Clare K Bryant M Roberts K Tahrani AA. Obesity and COVID-19: a call for action from people living with obesity. Lancet Diabetes Endocrinol (2020) 8(8):652–4. doi: 10.1016/s2213-8587(20)30236-9 PMC7836765 32653052

[27] Brown EM Clardy J Xavier RJ. Gut microbiome lipid metabolism and its impact on host physiology. Cell Host Microbe (2023) 31(2):173–86. doi: 10.1016/j.chom.2023.01.009 PMC10124142 36758518

[28] Seeberg KA Borgeraas H Hofsø D Småstuen MC Kvan NP Grimnes JO. Gastric bypass versus sleeve gastrectomy in type 2 diabetes: effects on hepatic steatosis and fibrosis: A randomized controlled trial. Ann Intern Med (2022) 175(1):74–83. doi: 10.7326/m21-1962 34843380

[29] Aron-Wisnewsky J Prifti E Belda E Ichou F Kayser BD Dao MC. Major microbiota dysbiosis in severe obesity: fate after bariatric surgery. Gut (2019) 68(1):70–82. doi: 10.1136/gutjnl-2018-316103 29899081 PMC7143256

[30] Arnoriaga-Rodríguez M Mayneris-Perxachs J Burokas A Pérez-Brocal V Moya A Portero-Otin M. Gut bacterial ClpB-like gene function is associated with decreased body weight and a characteristic microbiota profile. Microbiome (2020) 8(1):59. doi: 10.1186/s40168-020-00837-6 32354351 PMC7193372

[31] Yin J Li Y Han H Chen S Gao J Liu G. Melatonin reprogramming of gut microbiota improves lipid dysmetabolism in high-fat diet-fed mice. J Pineal Res (2018) 65(4):e12524. doi: 10.1111/jpi.12524 30230594

[32] Rau M Rehman A Dittrich M Groen AK Hermanns HM Seyfried F. Fecal SCFAs and SCFA-producing bacteria in gut microbiome of human NAFLD as a putative link to systemic T-cell activation and advanced disease. United Eur Gastroenterol J (2018) 6(10):1496–507. doi: 10.1177/2050640618804444 PMC6297934 30574320

[33] Wu TR Lin CS Chang CJ Lin TL Martel J Ko YF. Gut commensal Parabacteroides goldsteinii plays a predominant role in the anti-obesity effects of polysaccharides isolated from Hirsutella sinensis. Gut (2019) 68(2):248–62. doi: 10.1136/gutjnl-2017-315458 30007918

[34] Thingholm LB Rühlemann MC Koch M Fuqua B Laucke G Boehm R. Obese individuals with and without type 2 diabetes show different gut microbial functional capacity and composition. Cell Host Microbe (2019) 26(2):252–264.e210. doi: 10.1016/j.chom.2019.07.004 31399369 PMC7720933

[35] Adams LA Wang Z Liddle C Melton PE Ariff A Chandraratna H. Bile acids associate with specific gut microbiota, low-level alcohol consumption and liver fibrosis in patients with non-alcoholic fatty liver disease. Liver Int (2020) 40(6):1356–65. doi: 10.1111/liv.14453 32243703

[36] de Lastours V Poirel L Huttner B Harbarth S Denamur E Nordmann P. Emergence of colistin-resistant Gram-negative Enterobacterales in the gut of patients receiving oral colistin and neomycin decontamination. J Infect (2020) 80(5):578–606. doi: 10.1016/j.jinf.2020.01.003 31954100

[37] Péan N Le Lay A Brial F Wasserscheid J Rouch C Vincent M. Dominant gut Prevotella copri in gastrectomised non-obese diabetic Goto-Kakizaki rats improves glucose homeostasis through enhanced FXR signalling. Diabetologia (2020) 63(6):1223–35. doi: 10.1007/s00125-020-05122-7 PMC7228998 32173762

[38] Cryan JF O’Riordan KJ Cowan CSM Sandhu KV Bastiaanssen TFS Boehme M. The microbiota-gut-brain axis. Physiol Rev (2019) 99(4):1877–2013. doi: 10.1152/physrev.00018.2018 31460832

[39] Lavelle A Sokol H. Gut microbiota-derived metabolites as key actors in inflammatory bowel disease. Nat Rev Gastroenterol Hepatol (2020) 17(4):223–37. doi: 10.1038/s41575-019-0258-z 32076145

[40] Natividad JM Agus A Planchais J Lamas B Jarry AC Martin R. Impaired aryl hydrocarbon receptor ligand production by the gut microbiota is a key factor in metabolic syndrome. Cell Metab (2018) 28(5):737–749.e734. doi: 10.1016/j.cmet.2018.07.001 30057068

[41] Taleb S. Tryptophan dietary impacts gut barrier and metabolic diseases. Front Immunol (2019) 10:2113. doi: 10.3389/fimmu.2019.02113 31552046 PMC6746884

[42] Ma XH Liu JH Liu CY Sun WY Duan WJ Wang G. ALOX15-launched PUFA-phospholipids peroxidation increases the susceptibility of ferroptosis in ischemia-induced myocardial damage. Signal Transduct Target Ther (2022) 7(1):288. doi: 10.1038/s41392-022-01090-z 35970840 PMC9378747

[43] Miyamoto J Igarashi M Watanabe K Karaki SI Mukouyama H Kishino S. Gut microbiota confers host resistance to obesity by metabolizing dietary polyunsaturated fatty acids. Nat Commun (2019) 10(1):4007. doi: 10.1038/s41467-019-11978-0 31488836 PMC6728375

[44] de la Cuesta-Zuluaga J Mueller NT Álvarez-Quintero R Velásquez-Mejía EP Sierra JA Corrales-Agudelo V. Higher fecal short-chain fatty acid levels are associated with gut microbiome dysbiosis, obesity, hypertension and cardiometabolic disease risk factors. Nutrients (2018) 11(1):51. doi: 10.3390/nu11010051 30591685 PMC6356834

[45] Wu Q Sun L Hu X Wang X Xu F Chen B. Suppressing the intestinal farnesoid X receptor/sphingomyelin phosphodiesterase 3 axis decreases atherosclerosis. J Clin Invest (2021) 131(9):e142865. doi: 10.1172/jci142865 33938457 PMC8087211

[46] Patel R Santoro A Hofer P Tan D Oberer M Nelson AT. ATGL is a biosynthetic enzyme for fatty acid esters of hydroxy fatty acids. Nature (2022) 606(7916):968–75. doi: 10.1038/s41586-022-04787-x PMC9242854 35676490

[47] Pflimlin E Bielohuby M Korn M Breitschopf K Löhn M Wohlfart P. Acute and repeated treatment with 5-PAHSA or 9-PAHSA isomers does not improve glucose control in mice. Cell Metab (2018) 28(2):217–227.e213. doi: 10.1016/j.cmet.2018.05.028 29937376

[48] Marlatt KL Redman LM Beyl RA Smith SR Champagne CM Yi F. Racial differences in body composition and cardiometabolic risk during the menopause transition: a prospective, observational cohort study. Am J Obstet Gynecol (2020) 222(4):365.e361–365.e318. doi: 10.1016/j.ajog.2019.09.051 PMC7141969 31610152

[49] Fu Z Wu Q Guo W Gu J Zheng X Gong Y. Impaired insulin clearance as the initial regulator of obesity-associated hyperinsulinemia: novel insight into the underlying mechanism based on serum bile acid profiles. Diabetes Care (2022) 45(2):425–35. doi: 10.2337/dc21-1023 34880066

[50] Zhang X Zhang J Sun H Liu X Zheng Y Xu D. Defective phosphatidylglycerol remodeling causes hepatopathy, linking mitochondrial dysfunction to hepatosteatosis.y. Cell Mol Gastroenterol Hepatol (2019) 7(4):763–81. doi: 10.1016/j.jcmgh.2019.02.002 PMC6463126 30831319

[51] Zhao L Zhang F Ding X Wu G Lam YY Wang X. Gut bacteria selectively promoted by dietary fibers alleviate type 2 diabetes. Science (2018) 359(6380):1151–6. doi: 10.1126/science.aao5774 29590046

[52] Canfora EE Jocken JW Blaak EE. Short-chain fatty acids in control of body weight and insulin sensitivity. Nat Rev Endocrinol (2015) 11(10):577–91. doi: 10.1038/nrendo.2015.128 26260141

[53] West KA Kanu C Maric T McDonald JAK Nicholson JK Li JV. Longitudinal metabolic and gut bacterial profiling of pregnant women with previous bariatric surgery. Gut (2020) 69(8):1452–9. doi: 10.1136/gutjnl-2019-319620 PMC7398482 31964751

[54] Yoneshiro T Wang Q Tajima K Matsushita M Maki H Igarashi K. BCAA catabolism in brown fat controls energy homeostasis through SLC25A44. Nature (2019) 572(7771):614–9. doi: 10.1038/s41586-019-1503-x PMC6715529 31435015

[55] El-Zawawy HT El-Aghoury AA Katri KM El-Sharkawy EM Gad SMS. Cortisol/DHEA ratio in morbidly obese patients before and after bariatric surgery: Relation to metabolic parameters and cardiovascular performance. Int J Obes (Lond) (2022) 46(2):381–92. doi: 10.1038/s41366-021-00997-x 34725442

[56] Kolodziejczyk AA Zheng D Elinav E. Diet-microbiota interactions and personalized nutrition. Nat Rev Microbiol (2019) 17(12):742–53. doi: 10.1038/s41579-019-0256-8 31541197

[57] Fujiwara N Nakagawa H Enooku K Kudo Y Hayata Y Nakatsuka T. CPT2 downregulation adapts HCC to lipid-rich environment and promotes carcinogenesis via acylcarnitine accumulation in obesity. Gut (2018) 67(8):1493–504. doi: 10.1136/gutjnl-2017-315193 PMC6039238 29437870

[58] Zhao S Feng XF Huang T Luo HH Chen JX Zeng J. The association between acylcarnitine metabolites and cardiovascular disease in Chinese patients with type 2 diabetes mellitus. Front Endocrinol (Lausanne) (2020) 11:212. doi: 10.3389/fendo.2020.00212 32431666 PMC7214635

