# Supplementary material for: New mechanistic insights of anti-obesity by sleeve gastrectomy-altered gut microbiota and lipid metabolism
Source: Front Endocrinol (Lausanne). 2024 Feb 2;15:1338147. doi: 10.3389/fendo.2024.1338147 (PMC10875461; doi:10.3389/fendo.2024.1338147)
Supplement: Supplementary Figure 1 — (A) The dilution curves for Core genes; (B) The dilution curves for Pan genes; (C) The result for the Analysis of similarities (Anosim) between the two groups; (D) The result for the Adonis analysis between the two groups. [file Image_1.pdf]

A

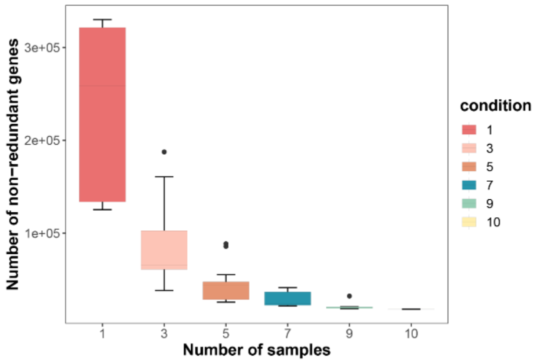

B

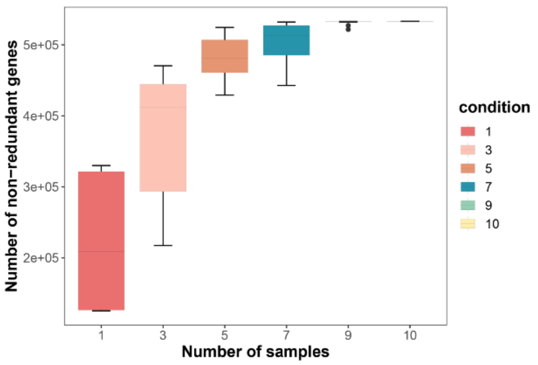

C

Method name	R statistic	p_value
bray_curtis	0.61	0.01

D

	Df	SumOfSqs	R2	F	Pr(>F)
Group	1	0.90	0.43	6.00	0.01
Residual	8	1.20	0.57		
Total	9	2.10	1		

Supplementary figure 1

(A) The dilution curves for Core genes; (B) The dilution curves for Pan genes; (C) The result for the Analysis of similarities (Anosim) between the two groups; (D) The result for the Adonis analysis between the two groups.

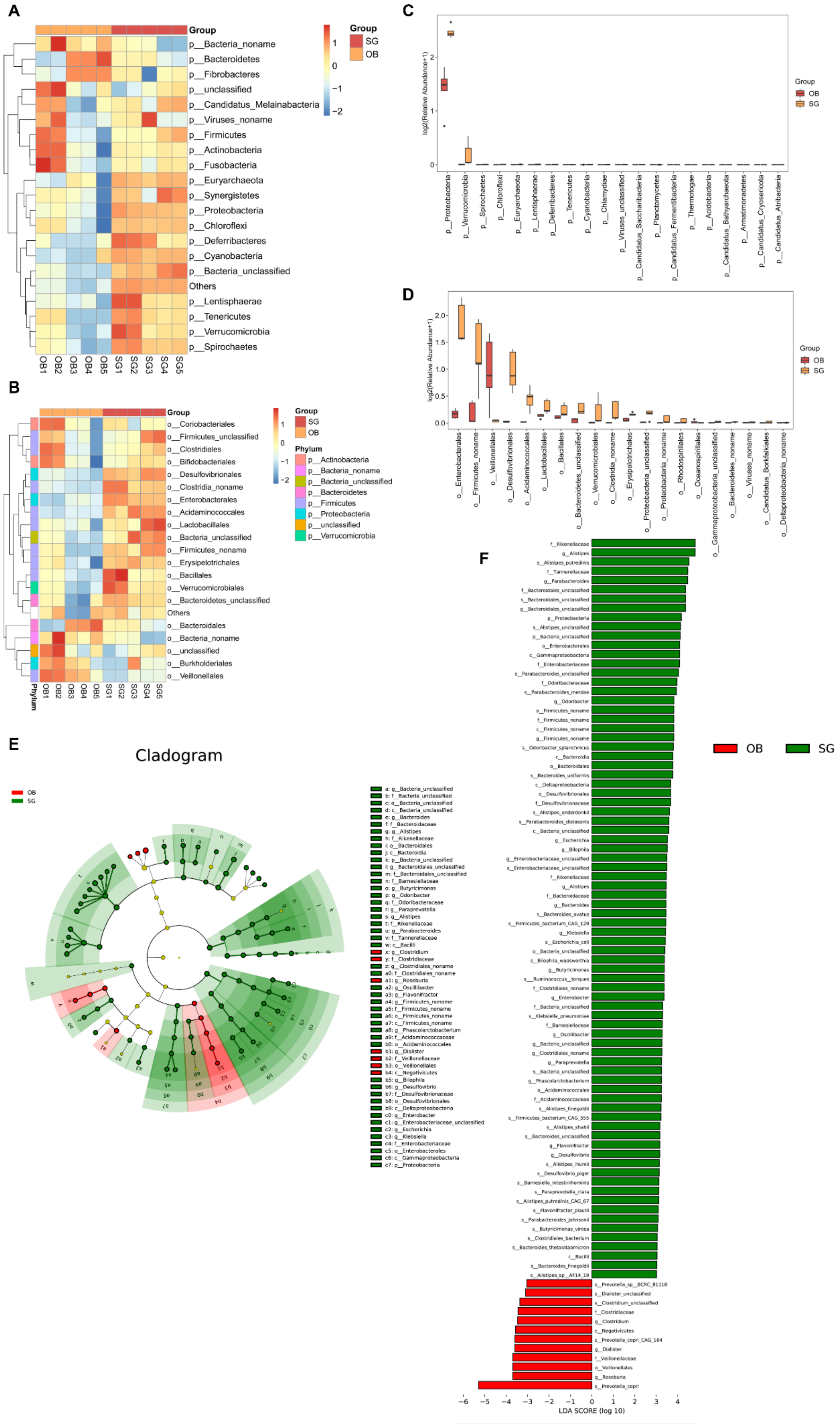

A
Group
SG
OB
p__Bacteria_noname
p__Bacteroidetes
p__Fibrobacteres
p__unclassified
p__Candidatus_Melainabacteria
p__Viruses_noname
p__Firmicutes
p__Actinobacteria
p__Fusobacteria
p__Euryarchaeota
p__Synergistetes
p__Proteobacteria
p__Chloroflexi
p__Deferribacteres
p__Cyanobacteria
p__Bacteria_unclassified
Others
p__Lentisphaerae
p__Tenericutes
p__Verrucomicrobia
p__Spirochaetes
OB1 OB2 OB3 OB4 OB5 SG1 SG2 SG3 SG4 SG5
B
Group
Phylum
o__Coriobacteriales
o__Firmicutes_unclassified
o__Clostridiales
o__Bifidobacteriales
o__Desulfovibrionales
o__Clostridia_noname
o__Enterobacterales
o__Acidaminococcales
o__Lactobacillales
o__Bacteria_unclassified
o__Firmicutes_noname
o__Erysipelotrichales
o__Bacillales
o__Verrucomicrobiales
o__Bacteroidetes_unclassified
Others
o__Bacteroidales
o__Bacteria_noname
o__unclassified
o__Burkholderiales
o__Veillonellales
p__Actinobacteria
p__Bacteria_noname
p__Bacteria_unclassified
p__Bacteroidetes
p__Firmicutes
p__Proteobacteria
p__unclassified
p__Verrucomicrobia
C
log2(Relative Abundance+1)
Group
OB
SG
p__Proteobacteria
p__Verrucomicrobia
p__Spirochaetes
p__Chloroflexi
p__Euryarchaeota
p__Lentisphaerae
p__Deferribacteres
p__Tenericutes
p__Cyanobacteria
p__Chlamydiae
p__Viruses_unclassified
p__Candidatus_Saccharibacteria
p__Planctomycetes
p__Candidatus_Fermentibacteria
p__Thermotogae
p__Acidobacteria
p__Candidatus_Bathyarchaeota
p__Armatimonadetes
p__Candidatus_Cryosericota
p__Candidatus_Atribacteria
D
log2(Relative Abundance+1)
o__Enterobacterales
o__Firmicutes_noname
o__Veillonellales
o__Desulfovibrionales
o__Acidaminococcales
o__Lactobacillales
o__Bacillales
o__Bacteroidetes_unclassified
o__Verrucomicrobiales
o__Clostridia_noname
o__Erysipelotrichales
o__Proteobacteria_unclassified
o__Proteobacteria_noname
o__Rhodospirillales
o__Oceanospirillales
o__Gammaproteobacteria_unclassified
o__Bacteroidetes_noname
o__Viruses_noname
o__Candidatus_Borkfalkiales
o__Deltaproteobacteria_noname
E
Cladogram
OB
SG
a: g__Bacteria_unclassified
b: f__Bacteria_unclassified
c: o__Bacteria_unclassified
d: c__Bacteria_unclassified
e: g__Bacteroides
f: f__Bacteroidaceae
g: g__Alistipes
h: f__Rikenellaceae
i: o__Bacteroidales
j: c__Bacteroidia
k: p__Bacteria_unclassified
l: g__Bacteroidales_unclassified
m: f__Bacteroidales_unclassified
n: f__Barnesiellaceae
o: g__Butyricimonas
p: g__Odoribacter
q: f__Odoribacteraceae
r: g__Paraprevotella
s: g__Alistipes
t: f__Rikenellaceae
u: g__Parabacteroides
v: f__Tannerellaceae
w: c__Bacilli
x: g__Clostridium
y: f__Clostridiaceae
z: g__Clostridiales_noname
a0: f__Clostridiales_noname
a1: g__Roseburia
a2: g__Oscillibacter
a3: g__Flavonifractor
a4: g__Firmicutes_noname
a5: f__Firmicutes_noname
a6: o__Firmicutes_noname
a7: c__Firmicutes_noname
a8: g__Phascolarctobacterium
a9: f__Acidaminococcaceae
b0: o__Acidaminococcales
b1: g__Dialister
b2: f__Veillonellaceae
b3: o__Veillonellales
b4: c__Negativicutes
b5: g__Bilophila
b6: g__Desulfovibrio
b7: f__Desulfovibrionaceae
b8: o__Desulfovibrionales
b9: c__Deltaproteobacteria
c0: g__Enterobacter
c1: g__Enterobacteriaceae_unclassified
c2: g__Escherichia
c3: g__Klebsiella
c4: f__Enterobacteriaceae
c5: o__Enterobacterales
c6: c__Gammaproteobacteria
c7: p__Proteobacteria
F
OB
SG
f__Rikenellaceae
g__Alistipes
s__Alistipes_putredinis
f__Tannerellaceae
g__Parabacteroides
f__Bacteroidales_unclassified
s__Bacteroidales_unclassified
g__Bacteroidales_unclassified
p__Proteobacteria
s__Alistipes_unclassified
p__Bacteria_unclassified
o__Enterobacterales
c__Gammaproteobacteria
f__Enterobacteriaceae
s__Parabacteroides_unclassified
f__Odoribacteraceae
s__Parabacteroides_merdae
g__Odoribacter
o__Firmicutes_noname
f__Firmicutes_noname
c__Firmicutes_noname
g__Firmicutes_noname
s__Odoribacter_splanchnicus
c__Bacteroidia
o__Bacteroidales
s__Bacteroides_uniformis
c__Deltaproteobacteria
o__Desulfovibrionales
f__Desulfovibrionaceae
s__Alistipes_onderdonkii
s__Parabacteroides_distasonis
c__Bacteria_unclassified
g__Escherichia
g__Bilophila
g__Enterobacteriaceae_unclassified
s__Enterobacteriaceae_unclassified
f__Rikenellaceae
g__Alistipes
f__Bacteroidaceae
g__Bacteroides
s__Bacteroides_ovatus
s__Firmicutes_bacterium_CAG_129
g__Klebsiella
s__Escherichia_coli
o__Bacteria_unclassified
s__Bilophila_wadsworthia
g__Butyricimonas
s__Ruminococcus_torques
f__Clostridiales_noname
g__Enterobacter
f__Bacteria_unclassified
s__Klebsiella_pneumoniae
f__Barnesiellaceae
g__Oscillibacter
g__Bacteria_unclassified
g__Clostridiales_noname
g__Paraprevotella
s__Bacteria_unclassified
g__Phascolarctobacterium
o__Acidaminococcales
f__Acidaminococcaceae
s__Alistipes_finegoldii
s__Firmicutes_bacterium_CAG_555
s__Alistipes_shahii
s__Bacteroides_unclassified
g__Flavonifractor
g__Desulfovibrio
s__Alistipes_ihumii
s__Desulfovibrio_piger
s__Barnesiella_intestinihominis
s__Paraprevotella_clara
s__Alistipes_putredinis_CAG_67
s__Flavonifractor_plautii
s__Parabacteroides_johnsonii
s__Butyricimonas_virosa
s__Clostridiales_bacterium
s__Bacteroides_thetaiotaomicron
c__Bacilli
s__Bacteroides_finegoldii
s__Alistipes_sp_AF14_19
s__Prevotella_sp_BCRC_81118
s__Dialister_unclassified
s__Clostridium_unclassified
f__Clostridiaceae
g__Clostridium
c__Negativicutes
s__Prevotella_copri_CAG_164
g__Dialister
f__Veillonellaceae
o__Veillonellales
g__Roseburia
s__Prevotella_copri
LDA SCORE (log 10)

Supplementary figure 2

(A) Changes of post-SG bacterial population at the phylum level presented as the heat map; (B) Changes of post-SG bacterial population at the order level presented as the heat map; (C) The TOP 20 differential microbial composition at the phylum level caused by SG, according to the P value; (D) The TOP 20 differential microbial composition at the order level caused by SG, according to the P value; (F) Cladogram of LDA Effect Size (LEfSe) for identifying species with significant differences in abundance of the SG group (LDA>3.0); (G) LDA score of LDA Effect Size (LEfSe) for identifying species with significant differences in abundance of the SG group (LDA>3.0).

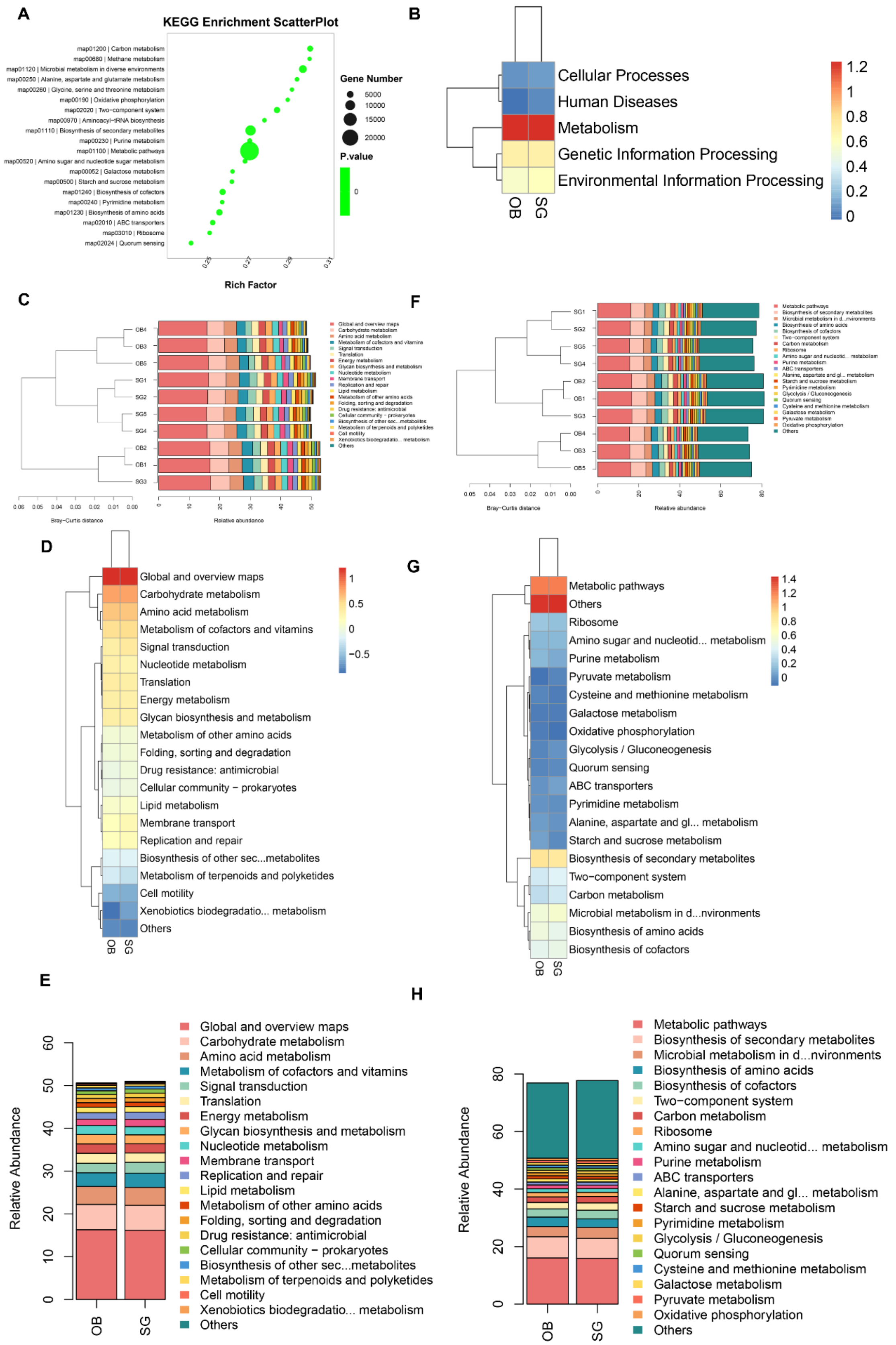
A
KEGG Enrichment ScatterPlot
map01200 | Carbon metabolism
map00680 | Methane metabolism
map01120 | Microbial metabolism in diverse environments
map00250 | Alanine, aspartate and glutamate metabolism
map00260 | Glycine, serine and threonine metabolism
map00190 | Oxidative phosphorylation
map02020 | Two−component system
map00970 | Aminoacyl−tRNA biosynthesis
map01110 | Biosynthesis of secondary metabolites
map00230 | Purine metabolism
map01100 | Metabolic pathways
map00520 | Amino sugar and nucleotide sugar metabolism
map00052 | Galactose metabolism
map00500 | Starch and sucrose metabolism
map01240 | Biosynthesis of cofactors
map00240 | Pyrimidine metabolism
map01230 | Biosynthesis of amino acids
map02010 | ABC transporters
map03010 | Ribosome
map02024 | Quorum sensing
Gene Number
5000
10000
15000
20000
P.value
0
0.25
0.27
0.29
0.31
Rich Factor
B
Cellular Processes
Human Diseases
Metabolism
Genetic Information Processing
Environmental Information Processing
OB
SG
1.2
1
0.8
0.6
0.4
0.2
0
C
OB4
OB3
OB5
SG1
SG2
SG5
SG4
OB2
OB1
SG3
Bray−Curtis distance
Relative abundance
F
SG1
SG2
SG5
SG4
OB2
OB1
SG3
OB4
OB3
OB5
Bray−Curtis distance
Relative abundance
D
Global and overview maps
Carbohydrate metabolism
Amino acid metabolism
Metabolism of cofactors and vitamins
Signal transduction
Nucleotide metabolism
Translation
Energy metabolism
Glycan biosynthesis and metabolism
Metabolism of other amino acids
Folding, sorting and degradation
Drug resistance: antimicrobial
Cellular community − prokaryotes
Lipid metabolism
Membrane transport
Replication and repair
Biosynthesis of other sec...metabolites
Metabolism of terpenoids and polyketides
Cell motility
Xenobiotics biodegradatio... metabolism
Others
OB
SG
1
0.5
0
−0.5
G
Metabolic pathways
Others
Ribosome
Amino sugar and nucleotid... metabolism
Purine metabolism
Pyruvate metabolism
Cysteine and methionine metabolism
Galactose metabolism
Oxidative phosphorylation
Glycolysis / Gluconeogenesis
Quorum sensing
ABC transporters
Pyrimidine metabolism
Alanine, aspartate and gl... metabolism
Starch and sucrose metabolism
Biosynthesis of secondary metabolites
Two−component system
Carbon metabolism
Microbial metabolism in d...nvironments
Biosynthesis of amino acids
Biosynthesis of cofactors
OB
SG
1.4
1.2
1
0.8
0.6
0.4
0.2
0
E
Relative Abundance
0
10
20
30
40
50
60
OB
SG
Global and overview maps
Carbohydrate metabolism
Amino acid metabolism
Metabolism of cofactors and vitamins
Signal transduction
Translation
Energy metabolism
Glycan biosynthesis and metabolism
Nucleotide metabolism
Membrane transport
Replication and repair
Lipid metabolism
Metabolism of other amino acids
Folding, sorting and degradation
Drug resistance: antimicrobial
Cellular community − prokaryotes
Biosynthesis of other sec...metabolites
Metabolism of terpenoids and polyketides
Cell motility
Xenobiotics biodegradatio... metabolism
Others
H
Relative Abundance
0
20
40
60
80
OB
SG
Metabolic pathways
Biosynthesis of secondary metabolites
Microbial metabolism in d...nvironments
Biosynthesis of amino acids
Biosynthesis of cofactors
Two−component system
Carbon metabolism
Ribosome
Amino sugar and nucleotid... metabolism
Purine metabolism
ABC transporters
Alanine, aspartate and gl... metabolism
Starch and sucrose metabolism
Pyrimidine metabolism
Glycolysis / Gluconeogenesis
Quorum sensing
Cysteine and methionine metabolism
Galactose metabolism
Pyruvate metabolism
Oxidative phosphorylation
Others

Supplementary figure 3

(A) The KEGG enrichment analysis of differential genes between groups presented as the scatterplot map; (B) Altered function of gut microbiota after SG based on KEGG database at the level1; (C, D, E) Altered function of gut microbiota after SG based on KEGG database at the KEGG level2; (F, G, H) Altered function of gut microbiota after SG based on KEGG database at the KEGG PathwayDefinition level.

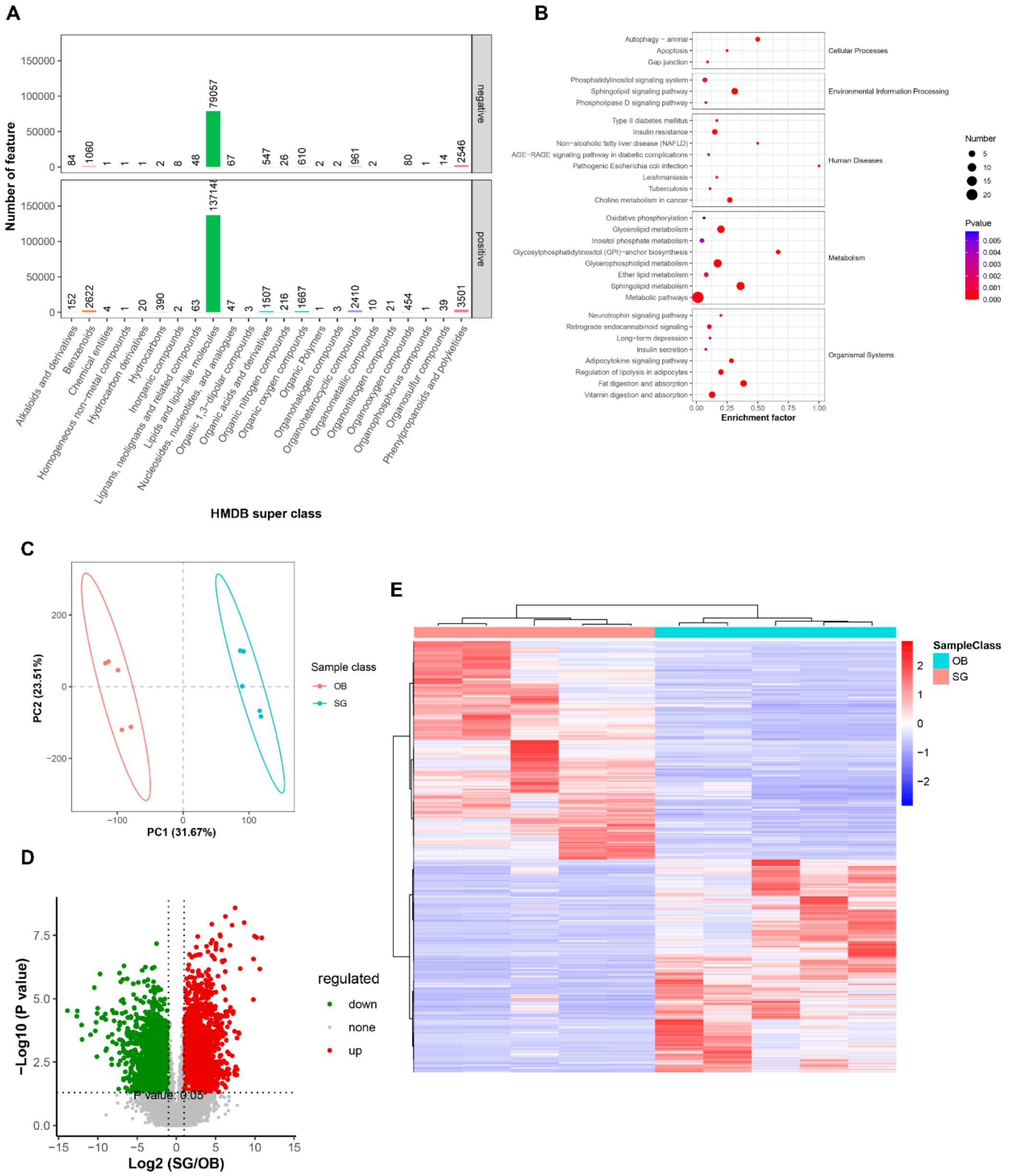

Supplementary figure 4

(A) the HMDB super class graph of the overall annotated gut metabolic features identified by MS1 based on the HMDB database; (B) the scatterplot graph of the overall annotated gut metabolic features identified by MS2 based on the KEGG database; (C) The partial least squares discriminant analysis (PLSDA) for gut metabolites between the two groups; (D) The volcano plot revealing the different gut metabolites between the two groups; (E) The heat maps revealing the different gut metabolites between the two groups.

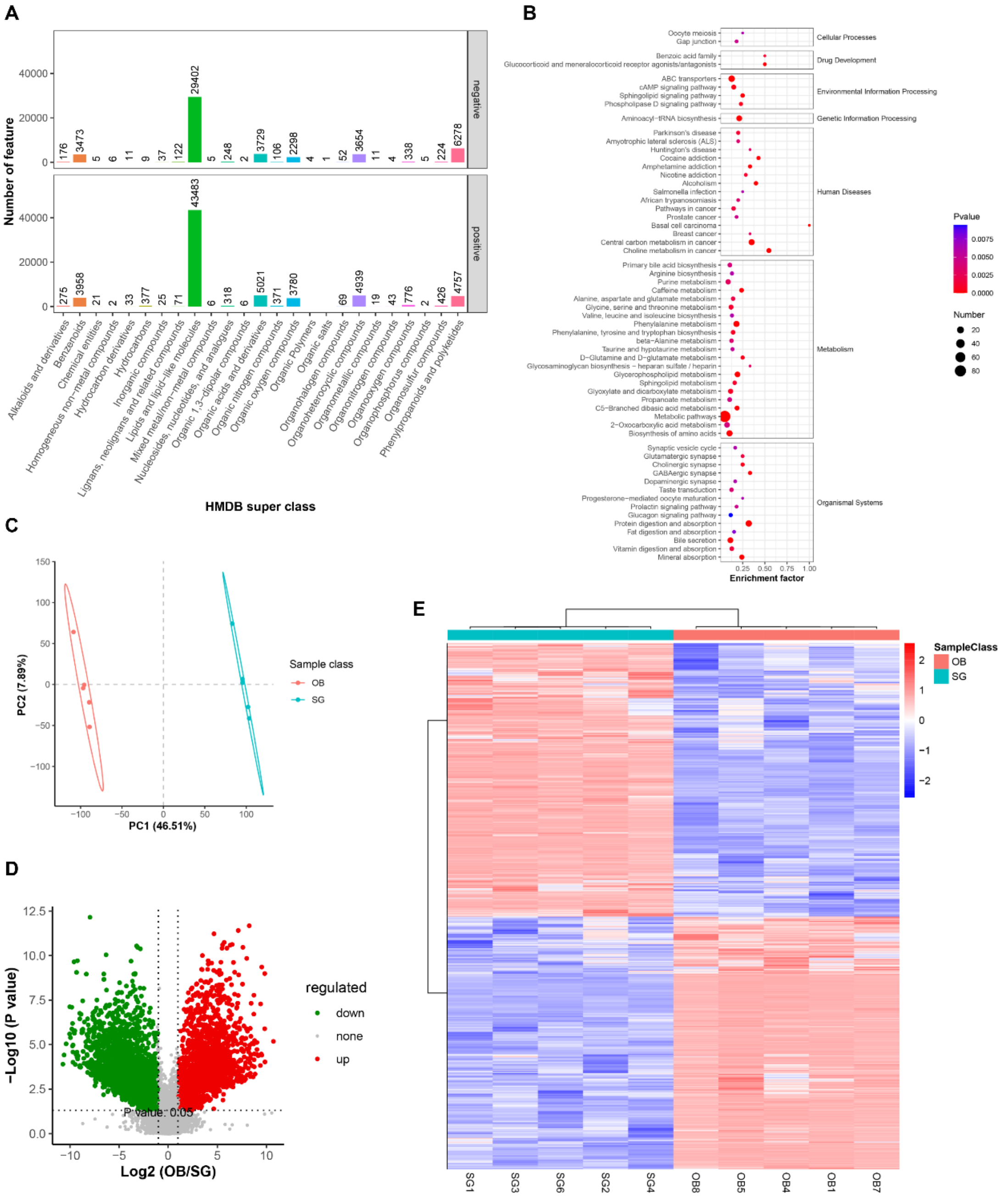

Supplementary figure 5

(A) the HMDB super class graph of the overall annotated serum metabolic features identified by MS1 based on the HMDB database; (B) the scatterplot graph of the overall annotated serum metabolic features identified by MS2 based on the KEGG database; (C) The partial least squares discriminant analysis (PLSDA) for serum metabolites between the two groups; (D) The volcano plot revealing the different serum metabolites between the two groups; (E) The heat maps revealing the different serum metabolites

between the two groups.

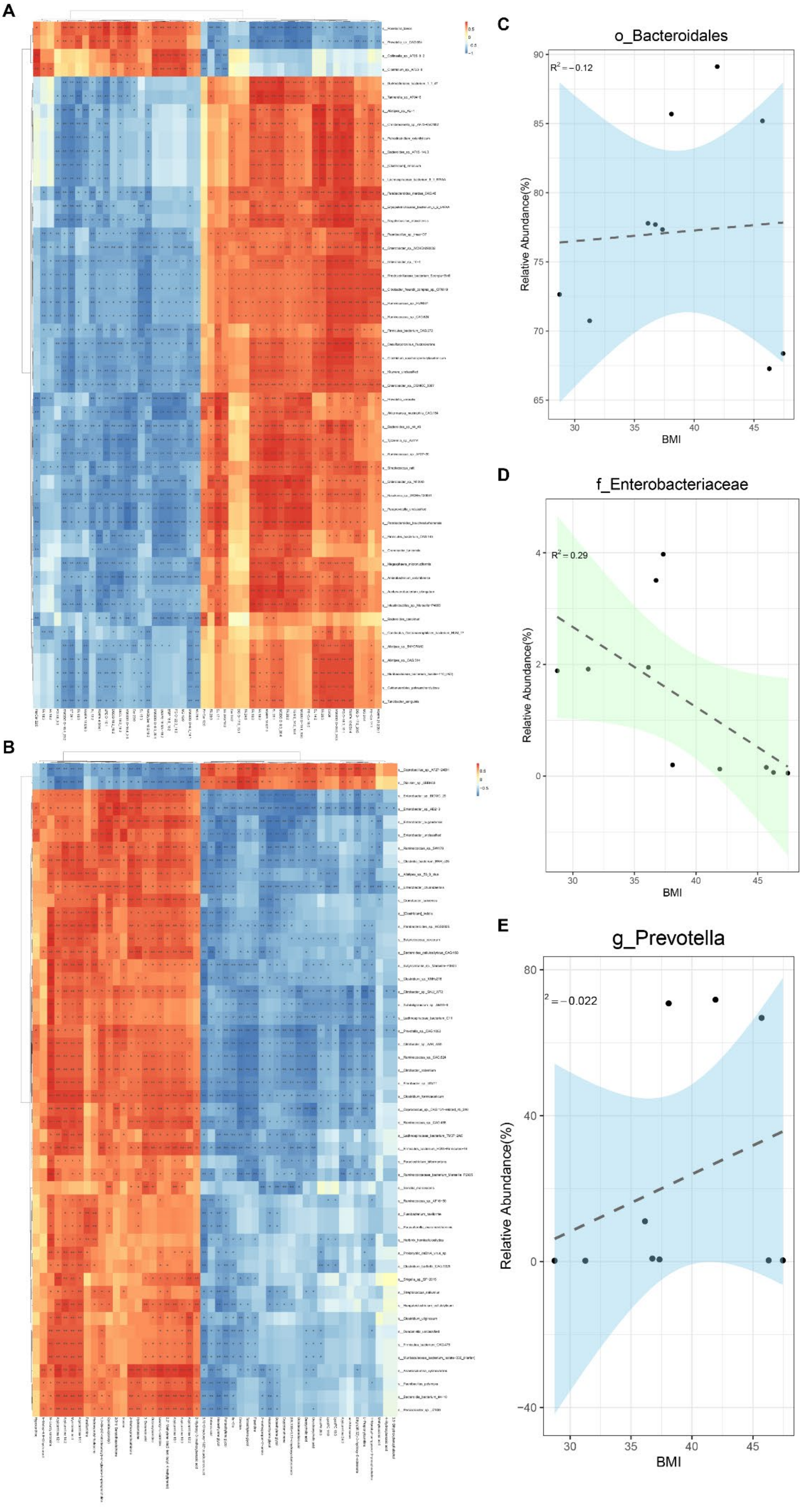
A
B
C
o_Bacteroidales
$R^2 = -0.12$
Relative Abundance(%)
BMI
D
f_Enterobacteriaceae
$R^2 = 0.29$
Relative Abundance(%)
BMI
E
g_Prevotella
$^2 = -0.022$
Relative Abundance(%)
BMI

Supplementary figure 6

(A) The correlation analysis between differential gut microbiota and gut differential metabolites; (B) The correlation analysis between differential gut microbiota and serum differential metabolites; (C) The linear correlation analysis between abundance of Bacteroidales and BMI; (D) The linear correlation analysis between abundance of Enterobacteriaceae and BMI; (E) The linear correlation analysis between abundance of Prevotella and BMI.
